# RNA polymerase II transcription attenuation at the yeast DNA repair gene *DEF1* is biologically significant and dependent on the Hrp1 RNA-recognition motif

**DOI:** 10.1093/g3journal/jkac292

**Published:** 2022-10-31

**Authors:** Maria E Amodeo, Shane P C Mitchell, Vincent Pavan, Jason N Kuehner

**Affiliations:** Department of Cancer Immunology & Virology, Dana Farber Cancer Institute, Boston, MA 02215, USA; Alzheimer Research Unit, MassGeneral Institute for Neurodegenerative Disease, Charlestown, MA 02129, USA; Department of Biology, Emmanuel College, Boston, MA 02115, USA; Department of Biology, Emmanuel College, Boston, MA 02115, USA

**Keywords:** RNA polymerase II, transcription termination, attenuation, *DEF1*, CPF-CF, NNS, Hrp1, RRM

## Abstract

Premature transcription termination (i.e. attenuation) is a potent gene regulatory mechanism that represses mRNA synthesis. Attenuation of RNA polymerase II is more prevalent than once appreciated, targeting 10–15% of mRNA genes in yeast through higher eukaryotes, but its significance and mechanism remain obscure. In the yeast *Saccharomyces cerevisiae*, polymerase II attenuation was initially shown to rely on Nrd1–Nab3–Sen1 termination, but more recently our laboratory characterized a hybrid termination pathway involving Hrp1, an RNA-binding protein in the 3′-end cleavage factor. One of the hybrid attenuation gene targets is *DEF1*, which encodes a repair protein that promotes degradation of polymerase II stalled at DNA lesions. In this study, we characterized the chromosomal *DEF1* attenuator and the functional role of Hrp1. *DEF1* attenuator mutants overexpressed Def1 mRNA and protein, exacerbated polymerase II degradation, and hindered cell growth, supporting a biologically significant *DEF1* attenuator function. Using an auxin-induced Hrp1 depletion system, we identified new Hrp1-dependent attenuators in *MNR2*, *SNG1*, and *RAD3* genes. An *hrp1-5* mutant (L205S) known to impair binding to cleavage factor protein Rna14 also disrupted attenuation, but surprisingly no widespread defect was observed for an *hrp1-1* mutant (K160E) located in the RNA-recognition motif. We designed a new RNA recognition motif mutant (*hrp1-F162W)* that altered a highly conserved residue and was lethal in single copy. In a heterozygous strain, *hrp1-F162W* exhibited dominant-negative readthrough defects at several gene attenuators. Overall, our results expand the hybrid RNA polymerase II termination pathway, confirming that Hrp1-dependent attenuation controls multiple yeast genes and may function through binding cleavage factor proteins and/or RNA.

## Introduction

As a testament to its biological utility, premature transcription termination (i.e. attenuation) is an ancient and widespread form of gene regulation, spanning all 3 domains of life and viruses (Kamieniarz-Gdula and Proudfoot 2019; Tatomer and Wilusz 2020; Rouvière *et al.* 2022). Instead of producing full-length mRNA, transcription attenuation results in a truncated RNA that encodes for an incomplete protein, lacks a coding sequence, and/or is unstable. Transcription attenuation was identified over 40 years ago in bacteria as a conditional mechanism that downregulates gene expression through the early release of RNA polymerase, causing incomplete mRNA synthesis (Turnbough 2019). Among the first attenuation targets to be characterized were mRNA genes that encoded amino acid biosynthesis enzymes (e.g. Trp operon). While initially thought to be rare in eukaryotes, attenuation has since been revealed to target thousands of protein-coding genes transcribed by RNA polymerase II (Pol II), including 10–15% of mRNA genes in the yeast *Saccharomyces cerevisiae* and higher eukaryotes (Neil *et al.* 2009; Kim and Levin 2011; Webb *et al.* 2014; Elrod *et al.* 2019; Tatomer *et al.* 2019).

The biological importance of eukaryotic attenuation is evidenced by a wide range of target gene functions, including transcriptional regulators, metabolic pathways, nucleotide biosynthesis enzymes, stress responses, signaling networks, and tumor suppressors (Kamieniarz-Gdula and Proudfoot 2019; Tatomer and Wilusz 2020; Rouvière *et al.* 2022). As a form of regulation, attenuation can fine-tune gene expression and/or limit mRNA synthesis to particular conditions and tissues (Wang *et al.* 2019). One of the first attenuation targets in yeast was identified in the *NRD1* gene, which encodes a termination factor that autoregulates its own expression (Arigo *et al.* 2006). Additional examples of attenuator-based autoregulation include 3′-end processing genes in yeast (*HRP1*, *PCF11*) and mammalian cells (*PCF11*, *CSTF77/CSTF3*) (Steinmetz *et al.* 2006; Kuehner and Brow 2008; Creamer *et al.* 2011; Luo *et al.* 2013; Grzechnik *et al.* 2015; Kamieniarz-Gdula *et al.* 2019; Wang *et al.* 2019). The importance of premature termination is further supported by attenuation defects linked to cancer, viral infection, developmental abnormalities, and neurodegeneration (Kamieniarz-Gdula and Proudfoot 2019; Tatomer and Wilusz 2020; Rouvière *et al.* 2022). The yeast 3′-end processing/termination protein Hrp1 is a focus of this study, and mutations in its human homolog HNRNPDL cause limb-girdle muscular dystrophy type 3 (Vieira *et al.* 2014).

Eukaryotic attenuation occurs through modulation of Pol II transcription termination. Pol II termination at the end of genes is coupled with RNA 3′-end processing and has been well studied in the model eukaryote yeast (*S. cerevisiae*) (Porrua and Libri 2015). Termination of Pol II mRNA synthesis typically involves recognition of polyadenylation (pA) sites and the Pol II C-terminal domain by cleavage factor (CF), and cleavage/polyadenylation factor (CPF), followed by recruitment of the Rat1 exoribonuclease. Initially, yeast attenuation was shown to rely on an alternative termination complex involving the RNA-binding proteins Nrd1 and Nab3 and RNA/DNA helicase Sen1 (NNS), the same pathway that terminates many noncoding RNAs (e.g. snRNAs, snoRNAs, cryptic unstable transcripts) (Arndt and Reines 2015). Our laboratory more recently characterized an attenuator in the DNA repair gene *DEF1* that relies on CF, CPF, and Sen1 without apparent involvement of Nrd1/Nab3, suggesting that yeast attenuation is not constrained to NNS (Whalen *et al.* 2018). We referred to the *DEF1* attenuation mechanism as “hybrid” termination, recognizing that others have documented similar CPF-CF and NNS crosstalk in 3′-end gene regions as part of a failsafe mechanism (Lemay and Bachand 2015). Interestingly, attenuation in higher eukaryotes relies on the Integrator complex, which similar to NNS, was first identified as an snRNA 3′-end processing factor (Mendoza-Figueroa *et al.* 2020; Kirstein *et al.* 2021). Integrator function during attenuation relies partly on subunits homologous to CPF proteins, akin to the yeast hybrid termination pathway.

We initially identified the yeast DNA repair gene *DEF1* as an attenuation target based on its usage of both promoter-proximal and promoter-distal pA sites (Graber *et al.* 2013; Whalen *et al.* 2018). *DEF1* encodes a protein essential for the degradation of Pol II stalled at UV-induced DNA lesions, which provides DNA repair factors with access to their substrate (Akinniyi and Reese 2021). Upon DNA damage, transcription shifts from producing noncoding attenuated RNA to full-length mRNA (Graber *et al.* 2013), likely as a means to upregulate *DEF1* expression and promote DNA repair. The mechanism of stress-induced *DEF1* attenuator readthrough remains unclear, particularly how hybrid termination may be modulated by DNA damage. In addition to the attenuator-based regulation of *DEF1*, further posttranslational control occurs whereby Def1 protein is restricted to the cytoplasm until UV-induced processing to give pr-Def1 stimulates nuclear import (Wilson *et al.* 2013). Despite its promoter-proximal location, the *DEF1* attenuator behaves unexpectedly, exhibiting strong dependence on a hybrid of CF/CPF and Sen1 components (Whalen *et al.* 2018). Consistent with the direct interaction of CF factor Hrp1 with *DEF1* attenuator RNA, mutations localized to a consensus pA site efficiency element (EE) and led to transcriptional readthrough that could be suppressed by *HRP1* overexpression. To test the importance of *DEF1* transcription attenuation the pA site EE mutation was combined with a pr-Def1 truncation mutation, which mimics UV-dependent processing and triggers Pol II degradation in the absence of stress (Wilson *et al.* 2013). The double mutant was more toxic than either single mutant alone, supporting a consequential function for *DEF1* transcription attenuation and a unique dual mechanism of regulation (Whalen *et al.* 2018).

Our studies have exposed an uncertainty regarding which attenuators confer biologically significant regulation, and if so, how they contribute to this effect. Few attenuators have been directly disrupted to test for consequences on cell fitness. Furthermore, our previous investigation was limited by the use of a high-copy *DEF1* plasmid, which may not accurately reflect its typical gene expression (Whalen *et al.* 2018). In this study, we used CRISPR mutagenesis to disrupt the chromosomal *DEF1* attenuator. The resulting *DEF1* overexpression enhanced toxicity when combined with pr-Def1 and exacerbated Pol II degradation, confirming the importance of attenuator-based regulation at the natural *DEF1* genomic locus. We also explored the mechanism by which CF factor Hrp1 promotes *DEF1* attenuation and whether Hrp1 contributes more broadly to attenuation via hybrid termination. Previous studies have revealed several lines of evidence consistent with wider Hrp1 function in attenuation: (1) Hrp1 autoregulates its mRNA expression via recognition of an attenuator in its 5′-UTR (Steinmetz *et al.* 2006; Kuehner and Brow 2008); (2) Hrp1 localizes to the 5′ ends of many mRNA genes (Tuck and Tollervey 2013); (3) there is a moderate correlation between the effect of *hrp1*-5 (L205S) and *sen1* (E1597K) mutants on transcription, consistent with Hrp1 influence on Sen1-dependent termination (Chen *et al.* 2017); and (4) *DEF1* attenuator readthrough occurs in *hrp1-1/nab4-1* (N167D, F179Y, P194H, Q265L), a temperature-sensitive mutant (Minvielle-Sebastia *et al.* 1998; Whalen *et al.* 2018). In this study, we further tested Hrp1 attenuator function via conditional depletion as well as point mutations that disrupted Hrp1 protein: protein and protein: RNA interfaces. We characterized new regions in the first Hrp1 RNA recognition motif (RRM) that are essential for attenuation as well as identified several new Hrp1-dependent attenuators, expanding the role of Hrp1 as a transcriptional regulator in yeast.

## Methods

### CRISPR mutagenesis of yeast DEF1 and HRP1 chromosomal loci

To create the *def1_1–530_*, *def1_atten_*, and *def1_1–530+atten_* mutant yeast strains, a CRISPR strategy was employed by cloning sgRNAs into p416-Cas9 (*URA3*) (Talkish *et al.* 2019) to target-specific regions of *DEF1* in yeast strain BY4742 or *HRP1* in yeast strain BY4742 *osTIR1::LEU2* for double-strand breaks. Following restriction digestion of p416-Cas9 with BaeI and phosphatase treatment, sgRNAs were cloned into the plasmid via Gibson cloning (New England Biolabs). Mutations were incorporated via yeast cotransformation of DNA repair templates with the p416-Cas9 CRISPR plasmid. Repair DNA for *DEF1* mutagenesis was constructed using a 3 primer PCR method (Ryan *et al.* 2016). Repair DNA for *HRP1* mutagenesis relied on PCR amplification using plasmid DNA template pKan-PCUP1-9myc-AID*(N) (N-terminal) (Morawska and Ulrich 2013) (a kind gift of the Ulrich lab). Following transformation, Ura+ candidates were plated on 5-FOA to counter-select against the p416-Cas9 plasmid. The *HRP1* C-terminal auxin-inducible degron (AID) tag was created through PCR and transformation of pKan-AID*-9myc and selection on G418 plates (Morawska and Ulrich 2013). Genomic DNA was purified from candidate colonies using a Masterpure Yeast DNA Purification Kit (Lucigen), followed by PCR, PCR clean-up, and sequencing to confirm desired mutations.

### QuikChange site-directed mutagenesis to create Hrp1 mutants

Hrp1 protein mutants (K160E, D193N, L205S, W168F, W168A, F162W, and F204W) were created using the QuikChange Lightening Kit based on manufacturer guidelines for primer design and PCR (Agilent). The PCR template was the pRS313-*HRP1* plasmid, which includes −500 to +1,848 of sequence relative to the *HRP1* + 1 ATG. The PCR product was digested with *Dpn1* enzyme prior to transformation into *E. coli* 5-alpha competent cells (New England Biolabs). The *E. coli* transformants were screened by plasmid purification and sequencing to confirm the presence of desired mutations.

### RT-PCR of Def1 mRNA

BY4742 strains containing *WT DEF1*, *def1_1–530_*, *def1_atten_*, or *def1_1–530+atten_* mutants were grown in YPAD until saturation at 25°C, diluted back in fresh media, and then grown at 25°C until OD_600_ ∼1.0. Pellets were obtained from 1.5 ODs of cells and frozen at −80°C. RNA was purified from cell pellets using the MasterPure Yeast RNA Purification Kit (Lucigen). After the RNA was isolated, mRNA was converted to cDNA using the OneTaq RT-PCR kit (New England BioLabs). A negative control that received no reverse transcriptase (-RT) was included to ensure that the final PCR product was RNA-dependent.

Briefly, total RNA (1 µg) from each sample was added to separate PCR tubes followed by 1 µl of 60 µM random primer. Nuclease-free water was added to bring each sample to a final volume of 4 µl. The RNA was denatured at 70°C for 5 min. Each +RT tube received 5 µl of M-MuLV Reaction Mix (2×) and 1 µl of the M-MuLV Enzyme Mix that contained the reverse transcriptase enzyme as well as dNTPs. The negative control received 5 µl of M-MuLV Reaction Mix (2×x) and 1 µl of nuclease-free water. All reactions were incubated at 25°C for 5 min, followed by a 1-h incubation at 42°C, and an enzyme inactivation at 80°C for 4 min. The reactions were diluted by adding 15 µl of nuclease-free water and stored at −20°C. Diluted cDNA (2 µl) was added to a new PCR tube and *DEF1* or 18S control primers were added to each tube along with OneTaq Hot Start 2X Master Mix (New England BioLabs) in a 25-µl reaction. The PCR cycle number was optimized for each product to maintain amplification in an appropriate linear range. PCR products (10 µl) were loaded into a 2% agarose gel along with the DNA reference ladder. The gel image was inverted, and band intensity was measured using Image Studio Software (LI-COR). The values were normalized by dividing the *DEF1* RT-PCR signal by the 18S loading control and comparing relative intensities.

### Western Blot analysis of Def1, Rpb1, and Hrp1 expression

BY4742 strains containing *WT DEF1*, *def1_1–530_*, or *def1_1–530+atten_* mutants were grown in YPAD until saturation at 25°C, diluted back in fresh media, and then grown until OD_600_ ∼1.0. For analysis of Rpb1 levels, strains were subsequently grown at 25°C or 39°C for an additional 2 h, followed by harvesting 1.5 ODs of cells and freezing pellets at −80°C. BY4742 *osTIR1::URA3*, HRP1-N-AID*-Myc + *HIS3*-marked *HRP1* wild-type and mutant alleles or empty vector + *LEU2*-marked lacZ reporter strains were grown overnight at 30°C in -Leu/-His media until saturation. Cultures were prepared by diluting back to OD_600_ = 0.8 in 25 ml of -Leu/-His media, followed by recovery in a 30°C shaking incubator for 2 h. The culture was split into separate cultures, with the addition of either 1 mM auxin (3-indoleacetic acid IAA, Sigma) or an equivalent volume of 100% ethanol solvent. Cultures were placed in the 30°C shaking incubator for 4 h, followed by harvesting 1.5 ODs and freezing pellets at −80°C.

Protein extracts were prepared using 2 M LiOAc and 0.4 M NaOH to permeabilize the yeast cell wall prior to extraction with 1× SDS-PAGE sample buffer (Zhang 2011). Protein extracts (10 µl) were loaded into a 12% SDS-PAGE gel, transferred to a PVDF membrane (Bio-Rad), and blocked in 5% milk/TBST for 1 h at room temperature. The membrane was cut between 75 and 50 kDa molecular weight markers. The upper portion was incubated overnight at 4°C with either rabbit polyclonal anti-Def1 antibody (diluted 1:5,000 in 1% milk/TBST, a kind gift of the Svejstrup lab), mouse anti-8WG16 primary antibody (diluted 1:2,000 in 1% milk/1× TBST; a kind gift of the Buratowski Lab), mouse anti-Myc primary antibody (diluted 1:2,000 in 1% milk/1× TBST; Santa Cruz Biotechnology; 9E10), or rabbit anti-Hrp1 primary antibody (diluted 1:15,000 in 1% milk/1× TBST; a kind gift of the Moore lab). The lower portion was incubated with a mouse monoclonal antiactin primary antibody (diluted 1:2,000 in 1% milk/TBST; Abcam ab8224) overnight at 4°C. The membrane was washed in 1× TBST and incubated with antirabbit or antimouse secondary antibody (diluted 1:15,000 in 1% milk/TBST; Jackson ImmunoResearch) for 1 h at room temperature. Target proteins were visualized using Clarity chemiluminescent substrate (Bio-Rad) and a C-DiGit Blot Scanner (LI-COR). The scanner images were uploaded into Image Studio Lite software (LI-COR) and the band intensities were quantified. The signal from Def1, Rpb1, or Hrp1 bands was divided by the signal of the actin bands to normalize each lane, which allowed for the comparison of protein levels across samples. Statistical testing was performed using Graphpad Prism version 9 for MacOS and Welch’s 2 sample *t*-test (*P ≤ 0.05, **P ≤ 0.01, ***P ≤ 0.001, ****P ≤ 0.0001, ns—not significant).

### Yeast spot test growth assay of def1 or hrp1 mutants

BY4742 strains containing *WT DEF1*, *def1_1–530_*, *def1_atten_*, or *def1_1–530+atten_* mutants were grown overnight at 25°C in YPAD until saturation. The pRS313-*HRP1* or pRS313-*hrp1* (HIS3) mutant plasmids were transformed into the shuffle strain BY4742 *hrp1*::KANMX [pRS316-*HRP1*] using a standard LiOAc procedure (Gietz and Schiestl 2007), and His+ transformants were grown overnight at 30°C in -His liquid media until saturation. BY4742 osTIR1::LEU2 yeast strains containing *HRP1 WT* and HRP1-N-AID*-Myc (N-terminal) or HRP1-C-AID*-Myc (C-terminal) were grown overnight at 30°C in YPAD until saturation. For all saturated cultures, dilutions were made to achieve an OD_600_ = 1.0. The strains were serially diluted from an OD_600_ of 1.0–0.001 across 4 rows. A replicator pin plater (Sigma) was used to transfer cells from a 96-well plate to the appropriate plates (YPAD, YPAD + 1 mM auxin, or 5-FOA) and incubated at 25°C, 30°C, 37°C, or 39°C for 3–5 days.

### Doubling time assay

BY4742 strains containing *def1_1–530_* or *def1_1–530+atten_* mutants were grown in YPAD until saturation at 25°C. Cultures were diluted to an OD_600_ = 0.15 and grown for a 2-h recovery at 30°C. Cultures were shifted to 37°C or 39°C, and OD_600_ measurements were recorded every hour for 6 h. The OD_600_ measurements were added to a scatterplot, and doubling times were calculated from exponential lines of best fit for data between 60 and 360 min. Statistical testing was performed using Graphpad Prism version 9 for MacOS and Welch’s 2 sample *t*-test (*P ≤ 0.05, **P ≤ 0.01, ***P ≤ 0.001, ****P ≤ 0.0001, ns—not significant).

### Integrated genome browser and heatmap analysis

The integrated genome browser (IGB) (Freese *et al.* 2016) was used to import and visualize the *S. cerevisiae* yeast genome, transcription start sites (TSS) (Pelechano *et al.* 2013), pA sites (Johnson *et al.* 2011), and proteins including RNA Pol II (Schaughency *et al.* 2014), Hrp1 (Tuck and Tollervey 2013), Nab3, and Nrd1 (Jamonnak *et al.* 2011). The peak height of pA sites and RNA-binding proteins near the 5′ end of genes was normalized to the Pol II signal in that region to account for differences in gene transcription rates. A ratio was calculated for pA, Pol II, Hrp1, Nrd1, and Nab3, by dividing the normalized value of each peak height by the value from the model NNS-dependent attenuator *NRD1*. These ratios were used to create a heatmap with 3 categories of site/factor enrichment: 0–0.67 (low), 0.67–1.5 (intermediate), and >1.5 (high). Based on the heatmap, candidates were chosen for additional study that showed high promoter-proximal enrichment (>1.5-fold) for Hrp1 protein and pA sites relative to *NRD1*.

### Gibson cloning of attenuator sequences into lacZ reporter plasmids

Attenuator candidates were amplified from yeast genomic DNA and cloned into pGAC24-noT-lacZ plasmids (LEU2) via the Gibson DNA Mastermix Assembly Kit (New England Biolabs). Briefly, primers were designed to amplify the following sequences relative to +1 ATG for *HDA2* (−283 to −1), *MNR2* (−225 to +116), *PTI1* (−201 to +195) *RAD3* (−139 to +175), *RPN4* (−264 to +98) and *SNG1* (−262 to +115), *SVF1* (−234 to +117), *TEC1* (−112 to +297), *UBC1* (−126 to +173), and *VTS1* (−187 to +82). Primers were designed using NEBuilder to contain appropriate homology to the pGAC24-noT-lacZ plasmid, which was digested with restriction enzyme XhoI and phosphatase-treated with Antarctic phosphatase (New England Biolabs) prior to assembly. Following transformation into 5-alpha competent cells (New England Biolabs), candidates were screened via colony PCR using GoTaq DNA Polymerase (Promega), and candidates containing inserts were sequenced.

### *Transformation of hrp1 mutants with the DEF1-, CYC1-*, *and HRP1-LacZ reporter genes*

The BY4742 *hrp1*::KANMX [pRS316-*HRP1*] shuffle strains transformed with pRS313-*HRP1*, *hrp1-K160E*, *hrp1-D193N*, *hrp1-L205S*, or *hrp1-W168F* were grown in YPAD liquid media and streaked onto 5-FOA plates to select for loss of the *URA3*-marked pRS316*-HRP1* plasmid. After 5-FOA counterselection, single colonies from each mutant were inoculated into YPAD media, followed by transformation with pGAC24-*DEF1*-lacZ, -*CYC1*-lacZ, or *HRP1*-lacZ (*LEU2*) reporter plasmids using a standard LiOAc procedure (Gietz and Schiestl 2007).

### LacZ reporter gene assays

BY4742 *hrp1*::KANMX shuffle strains containing pRS313-*HRP1 WT* or *hrp1* mutants as well as *LEU2*-marked attenuator-lacZ reporters were inoculated into -Leu/-His liquid media and grown overnight at 30°C until saturated. Cell density was measured via OD_600_ spectrophotometry (SpectraMax 190), and cells were diluted to OD_600_ = 0.15 and allowed to recover at 30°C for 2 h. Following recovery, cells were either processed immediately for β-Gal detection (see below), shifted to 37°C for 2 h, or treated with 1 mM auxin (3-indoleacetic acid IAA, Sigma) or ethanol solvent for 4 h. The density of each strain was measured, and the OD_600_ was adjusted between 0.2 and 0.6 if needed. Cell lysis was performed using 100 µl of culture and reporter enzyme activity was measured using the Yeast β-Galactosidase Assay Kit (Thermo Scientific). The absorbance at OD_420_ was measured every minute for 60 min to collect a kinetic reaction rate and slope. Slopes were gathered using a kinetic reaction window in a linear range with a strong *R*^2^ value. Relative β-galactosidase activity was calculated using the equation: [(OD420 Slope)/(0.1 ml) × OD600] = beta-galactosidase activity (Thibodeau *et al.* 2004). Statistical testing was performed using Graphpad Prism version 9 for MacOS and Welch’s 2 sample *t*-test or Welch’s ANOVA (*P ≤ 0.05, **P ≤ 0.01, ***P ≤ 0.001, ****P ≤ 0.0001, ns—not significant).

### PyMol structural analysis of the hrp1-F162W mutant

The Hrp1:RNA structure (2KM8) (Perez-Canadillas 2006) was accessed through the Protein Data Bank (Berman *et al.* 2000) and imported into PyMol (The PyMOL Molecular Graphics System, Version 2.0 Schrödinger, LLC). Residues were color-coded to highlight the Hrp1–F162 and RNA–A6 interaction. The PyMol mutagenesis wizard was used to test the impact of F162W, selecting the rotamer with the highest probability.

## Results

### The DEF1 attenuator mutant (def1_atten_) overexpresses mRNA and protein, exacerbating the cell toxicity of processed Def1 (def1_1–530_)

**Fig. 1.**
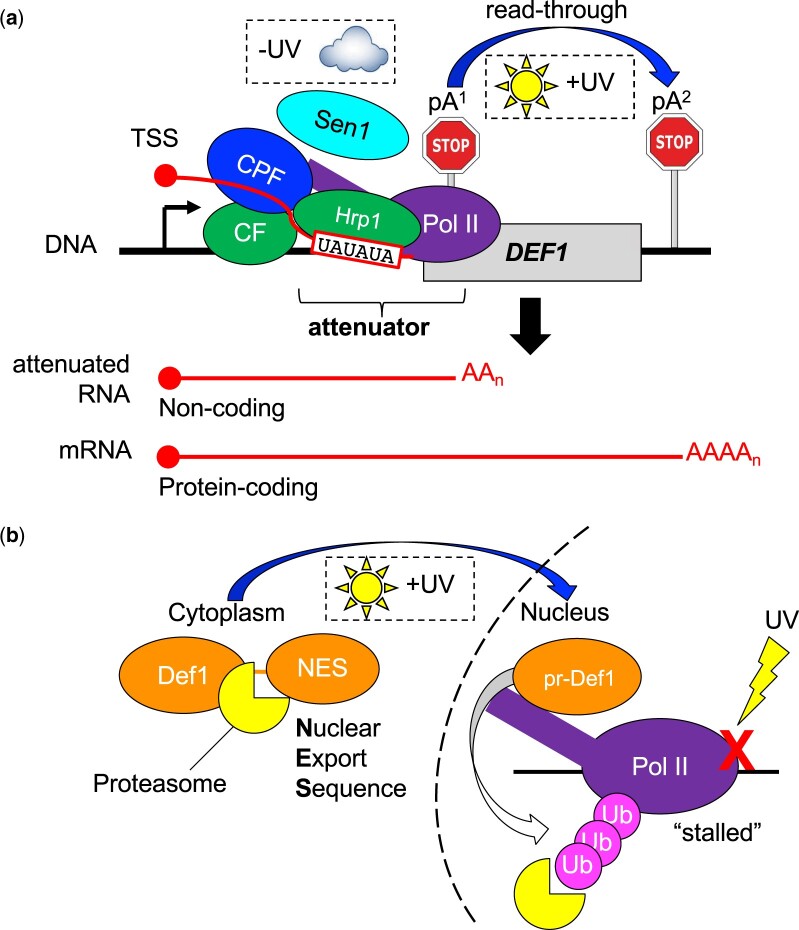
Expression of the yeast DNA repair gene *DEF1* is regulated by transcription attenuation and proteasome-mediated protein processing. a) Transcriptional regulation: in the absence of UV damage (−UV), Pol II transcription of *DEF1* undergoes premature termination (attenuation) via a hybrid CF/Hrp1/Sen1/CPF pathway, resulting primarily in noncoding RNA. In the presence of UV damage (+UV), Pol II readthrough of the attenuator results in bypass of pA^1^, leading to protein-coding mRNA and Def1 protein translation. b) Proteasomal regulation: in the absence of UV damage, Def1 protein remains cytoplasmic due to the presence of an NES. Following UV damage, the NES domain is removed by the proteasome, allowing processed Def1 (pr-Def1) to enter the nucleus. Nuclear pr-Def1 promotes ubiquitination (Ub) and degradation of Pol II stalled at UV-induced lesions (X), helping to promote DNA repair.

**Fig. 2.**
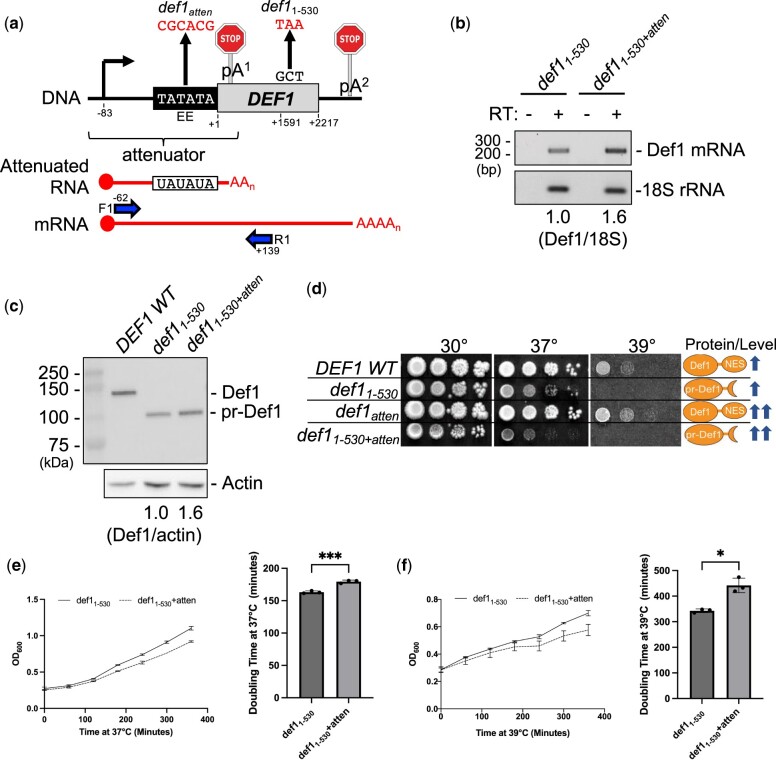
The def1 attenuator mutant (*def1_atten_*) overexpresses mRNA and protein, exacerbating the cell toxicity of processed Def1 (*def1_1–530_*). a) Schematic of *def1* mutants (*def1-atten* and *def1_1–530_*) along with RT-PCR primers (F1, R1, blue) used to detect the *DEF1* mRNA readthrough product. Note: the drawing is not to scale. The R1 primer is specific to longer mRNA and not attenuated RNA. DNA positions are numbered relative to the +1 start codon of the *DEF1* open reading frame. b) RT-PCR analysis of Def1 mRNA levels. 18S serves as a loading control for total RNA. Reverse Transcriptase (±RT) ensures that signal is dependent on RNA and not genomic DNA template. c) Western blot analysis of Def1 protein levels. Actin serves as a loading control for total protein. d) Spot test assay of *def1* mutants on solid plate media. Liquid cultures were grown to saturation at 25°C, serially diluted, spotted onto YPAD, and grown at the temperatures indicated for 3–5 days. e, f) Growth of *def1* mutants in liquid culture. Liquid yeast cultures were grown to saturation at 25°C, diluted back, and recovered to exponential phase prior to shifting to (e) 37°C or (f) 39°C for 6 h. Cell density was measured via OD_600_ every hour, and doubling times were calculated from exponential lines of best fit for data between 60 and 360 min. Error bars represent SD from 3 biological replicates. Asterisks indicate statistical significance by Welch’s 2 sample *t*-test (*P ≤0.05, **P≤0.01, ***P≤0.001, ****P≤0.0001, ns—not significant).

Our laboratory has previously demonstrated that Pol II transcription attenuation contributes to the regulation of *DEF1* gene expression and is biologically meaningful since attenuator mutants cause *DEF1* overexpression and cell toxicity (Whalen *et al.* 2018) (Fig. 1). However, the previous study was performed using a high-copy plasmid version of *DEF1*, which may not accurately reflect chromosomal gene expression. To assess the functional significance of the *DEF1* attenuator more reliably in vivo, we altered the *DEF1* attenuator locus using CRISPR-mediated genome editing. Based on previous mutagenesis results (Whalen *et al.* 2018), we created a mutation (*def1*_atten_) that changed the putative pA^1^ site EE (TATATA) to a nonconsensus element (CGCACG) (Fig. 2a). To mimic the truncated/processed version of Def1 protein (pr-Def1) that accumulates in the nucleus upon DNA Damage, we created a nonsense mutation (*def1*_1–530_) that changed the *DEF1* Ala531 codon to a stop codon and excluded the nuclear export sequence (NES). We also combined the mutations together (*def1*_1–530+atten_) to determine if the double mutant behaved any differently than the single mutants.

Consistent with our previous plasmid expression studies, the chromosomal *def1*_atten_ mutant increased Def1 mRNA expression ∼70% (1.7-fold) relative to a *DEF1* wild-type strain (Supplementary Fig. 1a), resulting in an 80% increase (1.8-fold) in Def1 protein expression (Supplementary Fig. 1, b and c). A similar increase in Def1 mRNA and protein expression (1.6-fold) was observed when *def1_atten_* was combined with the *def1*_1–530_ mutant (Fig. 2, b and c), which effectively truncated Def1 into a pr-Def1 protein (Fig. 2c). In a spot test growth assay, the *def1*_1–530_ mutant grew similarly to *DEF1* wild-type at 30°C but was temperature-sensitive at 37°C and 39°C (Fig. 2d). The temperature sensitivity of *def1*_1–530_ is consistent with activation/processing of Def1 and subsequent nuclear localization (Wilson *et al.* 2013). The *def1_atten_* mutant grew similarly to *DEF1* wild-type at all temperatures, presumably due to cytoplasmic localization of upregulated Def1 protein (Fig. 2d). The *def1*_1–530+atten_ double mutant grew similarly to *def1*_1–530_ at 30°C but was more temperature-sensitive than *def1*_1–530_ at 37°C, consistent with toxicity of nuclear Def1 overexpression (Fig. 2d). The exacerbation of temperature sensitivity in *def1*_1–530+atten_ vs *def1*_1–530_ was also observed when measuring growth in liquid culture. The *def1*_1–530+atten_ mutant took longer to grow compared with *def1*_1–530_, increasing the doubling time ∼10% upon shift to 37°C (Fig. 2e) and ∼30% upon shift to 39°C (Fig. 2f). These experiments confirm that attenuator-based regulation of the *DEF1* chromosomal locus is biologically significant, particularly in limiting pr-Def1 expression.

### Overexpression of processed Def1 (def1_1–530_) in a DEF1 attenuator mutant (def1_atten_) exacerbates proteasomal degradation of Pol II subunit Rpb1

**Fig. 3.**
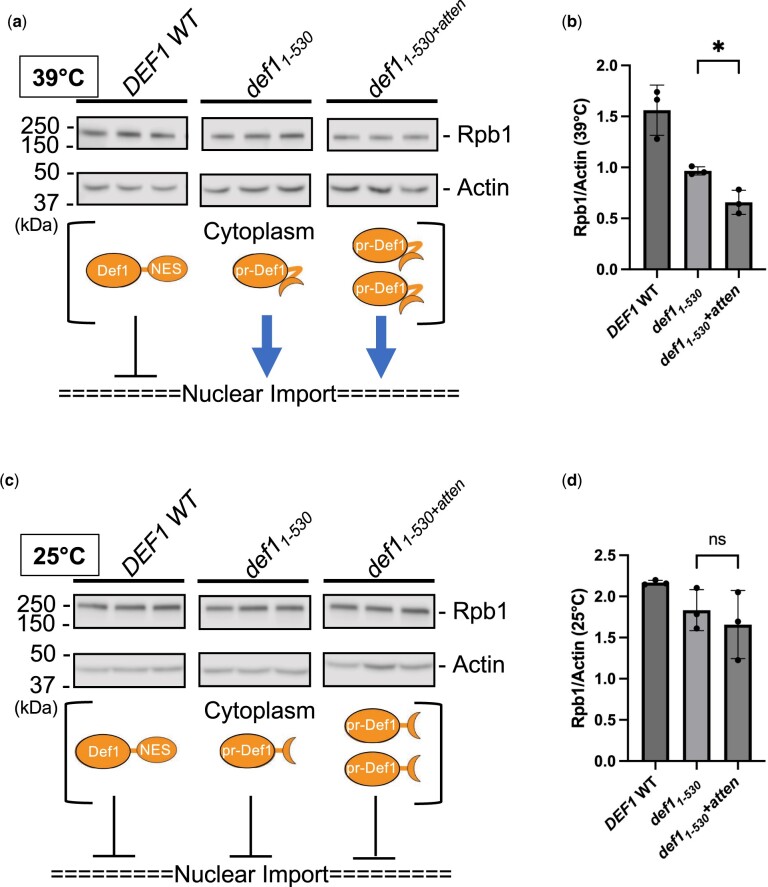
Overexpression of *def1*_1–530_ in a *def1* attenuator mutant (*def1*_atten_) exacerbates proteasomal degradation of Pol II subunit Rpb1. a, c) Western blot analysis of Rpb1 (Pol II) protein levels in *def1* mutants (biological triplicate samples). Liquid yeast cultures were grown to saturation at 25°C, diluted back, and recovered to exponential phase prior to shifting to (a) 39°C or (c) kept at 25°C for 2 h. Actin serves as a loading control for total protein. The schematic below the blot indicates the approximate Def1 protein expression level and localization (cytoplasm or nucleus) based on observed activity of the mutants. b, d) The average Rpb1 (Pol II) protein levels (normalized to actin) were quantified from the 3 biological replicates in (a) and (c), and error bars represent the SD. Asterisks indicate statistical significance by Welch’s 2 sample *t*-test.

One possible explanation for the enhanced toxicity of *def1*_1–530+atten_ vs *def1*_1–530_ is that overexpression of pr-Def1 in the nucleus leads to more acute proteasomal degradation of an essential protein. It has been observed that protein levels of the Pol II subunit Rpb1 decrease in *def1*_1–530_ grown at 37°C but not 25°, consistent with pr-Def1 triggering Pol II ubiquitination and degradation (Wilson *et al.* 2013). We investigated Rpb1 protein expression in our mutants and observed that Rpb1 levels were decreased in *def1*_1–530_ compared with *DEF1* wild-type at elevated temperature (39°C) (Fig. 3, a and b). There was a further reduction in Rpb1 protein in *def1*_1–530+atten_ compared with *def1*_1–530_ at 39°C, which correlates with the magnitude of temperature-sensitivity observed in solid and liquid media (Fig. 2, d and e). The was no significant difference in Rpb1 protein levels when comparing *def1*_1–530_ and *def1*_atten_ mutants grown at the permissive temperature of 25°C (Fig. 3, c and d). These data expand on the biological significance of the *DEF1* attenuator and are consistent with its regulation of Def1 mRNA/protein expression, perhaps to prevent Pol II degradation and transcriptome disruption.

### Hrp1 function in attenuator recognition is dependent on its interaction with CFIA and RNA to a variable extent depending on the Pol II terminator

**Fig. 4.**
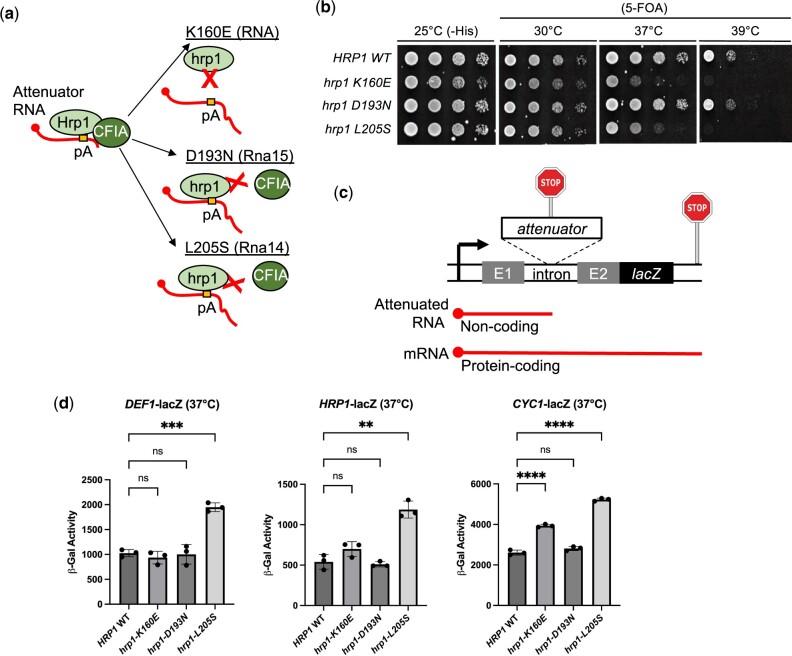
Hrp1 function in attenuator recognition is dependent on its interaction with CFIA and RNA and varies based on the Pol II terminator. a) Schematic of Hrp1 activity and *hrp1* mutants expected to disrupt interaction with RNA (K160E), CFIA protein Rna15 (D193N), or CFIA protein Rna14 (L205S). b) Spot test growth assay of *hrp1* mutants. A yeast shuffle strain [*hrp1::KANMX*, pRS316-*HRP1* (*URA3*)] was transformed with *HIS3*-marked plasmids containing *hrp1* mutants prior to 5-FOA shuffling. Serial dilutions were spotted on plates and grown 3 days at indicated temperatures. c) Schematic of attenuator-lacZ reporter gene system. d) Attenuator functionality assays using a lacZ reporter gene with *hrp1* mutants. Yeast strains bearing *HRP1* WT or *hrp1* mutant plasmids were transformed with lacZ reporter genes containing *DEF1* or *HRP1* attenuators. The *CYC1* terminator serves as a control for hybrid termination. Overnight cultures were grown to saturation at 30°C and recovered to exponential phase followed by a 2-h shift to nonpermissive temperature (37°C). Cells were lysed and β-galactosidase activity was measured to detect attenuator readthrough. Error bars represent SD of 3 biological replicates. Asterisks indicate statistical significance by Welch’s ANOVA.

To further understand the mechanism of *DEF1* attenuator recognition, we expanded our study of *cis*-acting elements and *trans*-acting factors to additional model genes. Our laboratory has previously observed that mutations in the Hrp1 protein and its putative binding site within an RNA EE both disrupt *DEF1* attenuation (Whalen *et al.* 2018), but the exact function of Hrp1 in this Pol II termination process remains unclear. Hrp1 has at least 2 critical roles in 3′-end processing/termination: (1) binding RNA and (2) binding CFIA proteins Rna15 and Rna14 (Leeper *et al.* 2010). To distinguish the importance of these roles in attenuation, we tested the effect of 3 *hrp1* mutants that impair various aspects of its function: (1) K160E (*hrp1-1*), defective in Hrp1-RNA binding (Perez-Canadillas 2006); (2) D193N, predicted to disrupt Hrp1-Rna15 binding (Leeper *et al.* 2010); and (3) L205S (*hrp1-5*), defective in Hrp1-Rna14 binding (Barnwal *et al.* 2012) (Fig. 4a). Since the *hrp1*-*D193N* and *L205S* mutants are still able to bind RNA in vitro, they were used to test the importance of the Hrp1–CFIA protein interaction in vivo.

To confirm that the *hrp1* mutants were behaving as expected in our strain background, we performed a spot test assay with yeast containing mutant plasmids and measured growth before or after shuffling out an *HRP1* wild-type plasmid on 5-FOA media. At the permissive temperature of 30°C, mutant and wild-type strains grew at similar levels after 5-FOA shuffling (Fig. 4b). The *hrp1*-*K160E* and *L205S* mutants grew more slowly than wild-type at elevated temperature (37°C) and exhibited lethality at 39°C, consistent with previous findings documenting their temperature-sensitivity (Perez-Canadillas 2006; Barnwal *et al.* 2012). The *hrp1-D193N* mutant grew indistinguishably from wild-type and was not temperature-sensitive, similar to what was observed previously for *hrp1-D193R* (Leeper *et al.* 2010).

To determine if the *hrp1* mutants altered Pol II termination, we used a lacZ reporter gene system in which terminator readthrough can be measured by increased β-galactosidase activity (Whalen *et al.*, 2018) (Fig. 4c). Our reporter genes contained either upstream attenuator regions from *DEF1* and *HRP1* genes or the downstream terminator from the *CYC1* gene, which have all been shown to rely on Hrp1 (Whalen *et al.* 2018). The *hrp1-L205S* mutant exhibited the most pronounced termination defect, increasing readthrough ∼2-fold compared with wild-type for all 3 reporter genes (Fig. 4d). This result is consistent with the Hrp1–Rna14 interaction supporting attenuation across a broad range of Pol II terminators. In contrast, the *hrp1-D193N* mutant did not affect termination for any gene analyzed, consistent with its lack of a temperature-sensitive growth defect. The *hrp1*-*K160E* mutant exhibited a mild gene-specific termination defect, increasing readthrough ∼1.5-fold for *CYC1* but no significant consequence for *HRP1* or *DEF1*. Overall, these data suggest that Hrp1 may function in attenuation based on its ability to bind RNA and CFIA proteins in a gene-specific manner.

### The hrp1 RRM1 mutants W168A, F162W, and F204W are lethal and defective for attenuation

**Fig. 5.**
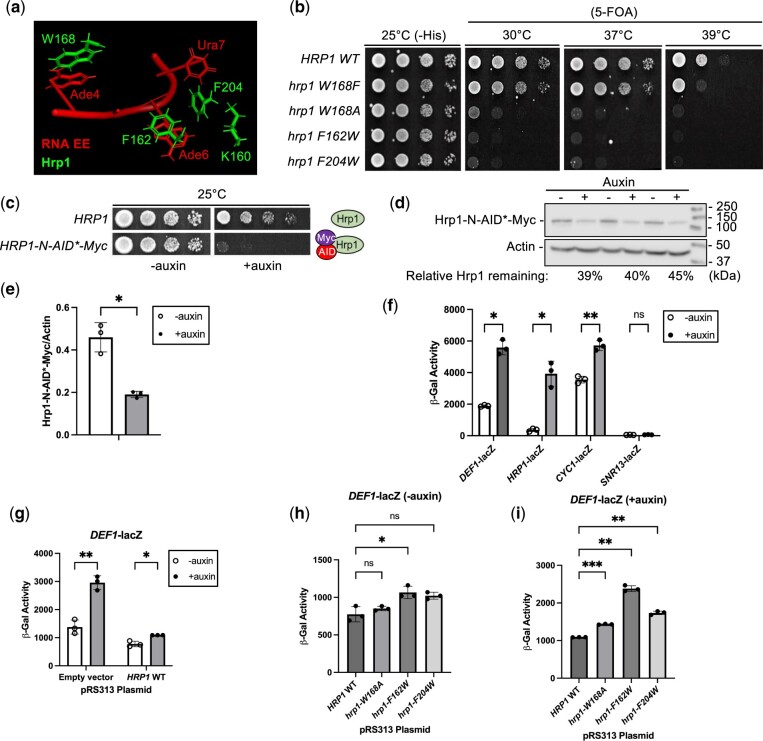
The hrp1 RRM1 mutants W168A, F162W, and F204W are lethal and defective for attenuation. a) Protein–RNA interface for the Hrp1-EE complex (structure 2KM8; PyMOL). Relevant Hrp1 side chains (green) and RNA bases (red) are represented in stick format. b) Spot test growth assay of *hrp1* mutants. A yeast shuffle strain [*hrp1::KANMX*, pRS316-*HRP1* (*URA3*)] was transformed with plasmids containing *hrp1* mutants prior to 5-FOA shuffling. Serial dilutions were spotted on plates and grown 3 days at indicated temperatures. c) Yeast strains containing *osTIR1* and WT (*HRP1)* or an N-terminal degron tag (HRP1-N-AID*-Myc) were grown on YPAD ± auxin inducer (1 mM) for 3 days. d) Western blot analysis of Hrp1 depletion. Hrp1 degron strains (in biological triplicate) were grown in YPAD until exponential phase, followed by treatment with auxin (1 mM) or ethanol solvent control for 4 h. Hrp1 was detected from protein extracts with an anti-Myc antibody and normalized to actin as a loading control. e) Average Hrp1 protein levels were quantified from 3 biological replicates, and error bars represent SD. f) The Hrp1 degron strain (HRP1-N-AID*-Myc) was transformed with lacZ reporter genes and grown in selective media ± auxin for 4 h. Cells were lysed and β-galactosidase activity was measured to detect attenuator readthrough. Error bars represent SD of 3 biological replicates. g–i) The Hrp1 degron strain containing *DEF1*-lacZ was transformed with pRS313 plasmids containing empty vector, *HRP1* WT, or *hrp1* mutants. Auxin treatment and β-gal assays were performed as in (f). Asterisks indicate statistical significance by Welch’s 2 sample *t*-test.

The minimal terminator readthrough defect for the *hrp1-K160E* mutant was surprising given that RNA EE mutants in *DEF1*, *HRP1*, and *CYC1* disrupt 3′-end formation (Russo *et al.* 1993; Whalen *et al.* 2018). The *hrp1-1* mutant (K160E) was initially identified as a conditional allele with defects in pA tail length (Kessler *et al.* 1997). Guided by an Hrp1-RNA-CFIA structural model, we targeted Hrp1 amino acids W168, F162, and F204 for mutagenesis. These amino acid residues are also located in the first Hrp1 RRM and contribute to the EE RNA interface (Ade4, Ade6, or Ura7) (Perez-Canadillas 2006; Barnwal *et al.* 2012) (Fig. 5a).

To test the effect of our new *hrp1* RRM mutants on cell growth, we performed a spot test assay with mutant plasmids either before or after shuffling out an *HRP1* wild-type plasmid on 5-FOA media. The W → F and F → W substitutions were chosen to be relatively conservative (given the aromatic properties of both Phe and Trp) and selectively disrupt RNA binding without global misfolding of the protein. The *hrp1-W168F* mutant was least consequential, growing similarly to wild-type at all temperatures, aside from mild sensitivity at 37°C and 39°C (Fig. 5b). Surprisingly, when the *hrp1-W168F* mutant was tested in the context of the *DEF1*-, *HRP1*-, and *CYC1*-lacZ reporter genes, it suppressed readthrough to a slight extent (Supplementary Fig. 2). These data suggest that *hrp1*-*W168F* may produce a more efficient attenuator, perhaps via better binding to RNA EEs. The *hrp1*-*W168A*, *F162W*, and *F204W* mutants were lethal on 5-FOA at all temperatures tested (Fig. 5b), preventing us from studying their function in the original shuffle strain.

To assess the effects of the lethal *hrp1* mutants (*F162W*, *F204W*, and *W168A*) in vivo, we needed a rapid method to remove wild-type Hrp1 before its loss caused cell death. A classical approach for studying essential genes is the use of conditional temperature-sensitive (*ts*) alleles to inactivate a protein. However, there are limitations to *ts* alleles, including the fact that a temperature shift from 25°C to 37°C induces a potentially confounding heat-shock response. For example, the *FKS2* attenuator exhibits Pol II readthrough during heat shock, activating genes in the yeast cell wall integrity pathway (Kim and Levin 2011). Furthermore, some conditional *hrp1* mutants exhibit near wild-type growth at a permissive temperature of 25°C but still exhibit underlying readthrough defects (Kuehner and Brow 2008), making it harder to detect differences above this background leakiness.

An alternative method to *ts* alleles for conditional protein depletion is an AID system (Nishimura *et al.* 2009). This approach relies on augmenting a protein of interest with a degron tag. Upon addition of an auxin inducer, the protein–AID fusion interacts with an E3 ubiquitin ligase complex, resulting in poly-ubiquitination of the AID and proteasomal degradation. An advantage of an AID vs a *ts* allele is that it can be performed at a constant temperature that avoids stress responses. We introduced an AID-Myc tag at the N-terminus or C-terminus of the *HRP1* chromosomal gene locus. The N-terminal tag (HRP1-N-AID*-Myc) appeared successful because the strain grew similarly to a wild-type untagged strain at 25°C without auxin but exhibited toxicity with auxin (Fig. 5c). The Hrp1-N-AID*-Myc protein was depleted ∼60% after a 4-h auxin treatment (Fig. 5, d and e). A C-terminal tag strain was auxin-sensitive at 25°C, but it was more sensitive than an untagged strain at 37°C −auxin (Supplementary Fig. 3a). Furthermore, the Hrp1 C-terminal degron strain exhibited readthrough of the *DEF1*-lacZ reporter gene even in the absence of auxin (Supplementary Fig. 3b**)**. The abnormal behavior of the Hrp1 C-terminal degron strain suggested a tag-induced protein folding defect so it was not studied further.

To test the consequences of Hrp1-N-AID*-Myc depletion on attenuation, we measured the lacZ activity of several mRNA termination reporters and an NNS-dependent snoRNA gene (*SNR13*). Consistent with previously observed defects in *hrp1* ts mutants (Whalen *et al.* 2018), Hrp1 depletion increased readthrough for *DEF1* (3.0-fold), *HRP1* (10.7-fold), and *CYC1* (1.6-fold) terminators, albeit to varying extents (Fig. 5f). No significant defect was observed for the *SNR13* snoRNA terminator after Hrp1 depletion, consistent with its dependence on the NNS pathway (Fig. 5f) (Whalen *et al.* 2018). Hrp1 depletion was also AID-specific, with no readthrough observed for reporters in untagged *HRP1* strains treated with auxin (Supplementary Fig. 4). These data indicate that premature termination at some attenuator regions is strongly Hrp1-dependent, while other Pol II terminators operate more independently of Hrp1.

To further characterize the Hrp1-dependence of the *DEF1* and *HRP1* attenuators, we monitored lacZ reporter activity in Hrp1 degron strains containing plasmid-based copies of *HRP1* wild-type, lethal *hrp1* RRM mutants (*F162W*, *F204W*, and *W168A*), or an empty vector control. The Hrp1 degron strain with empty vector exhibited readthrough defects for *DEF1* ± auxin (2.1-fold), and the readthrough defect was less severe (1.4-fold) upon plasmid addition of *HRP1* wild-type (Fig. 5g). When comparing *hrp1* RRM mutants vs *HRP1* wild-type there was a slight *DEF1* readthrough defect (1.4-fold) in *F162W* even in the absence of auxin, consistent with a dominant-negative effect of the mutation (Fig. 5h). The *DEF1* readthrough defect in *hrp1-F162W* was exacerbated in the presence of auxin (2.2-fold), with *W168A* and *F204W* mutants less defective (1.3- to 1.6-fold, respectively) (Fig. 5i). There were stronger readthrough defects in all *hrp1* mutants for the *HRP1* attenuator and *CYC1* terminator in both the absence and presence of auxin, and *F162W* was again most defective (Supplementary Fig. 5). Overall, these data suggest that conserved Hrp1 RRM residues W168, F162, and F204 all support optimal attenuator function, but F162 contributes a predominant role for this gene subset.

### Heatmap analysis of Hrp1 and pA promoter-proximal enrichment helps identify new attenuators, some of which are Hrp1-dependent

To extend our understanding of premature Pol II termination beyond the *DEF1* and *HRP1* model genes, we explored published datasets for evidence of other genes bearing attenuation signatures. We used the IGB to align the relative genome occupancy of Pol II (Rpb1) (Schaughency *et al.* 2014), Hrp1 (Tuck and Tollervey 2013), and Nrd1 and Nab3 proteins (Jamonnak *et al.* 2011) as well as RNA TSS (Pelechano *et al.* 2013) and pA sites (Johnson *et al.* 2011) near the 5′ end of genes.

**Fig. 6.**
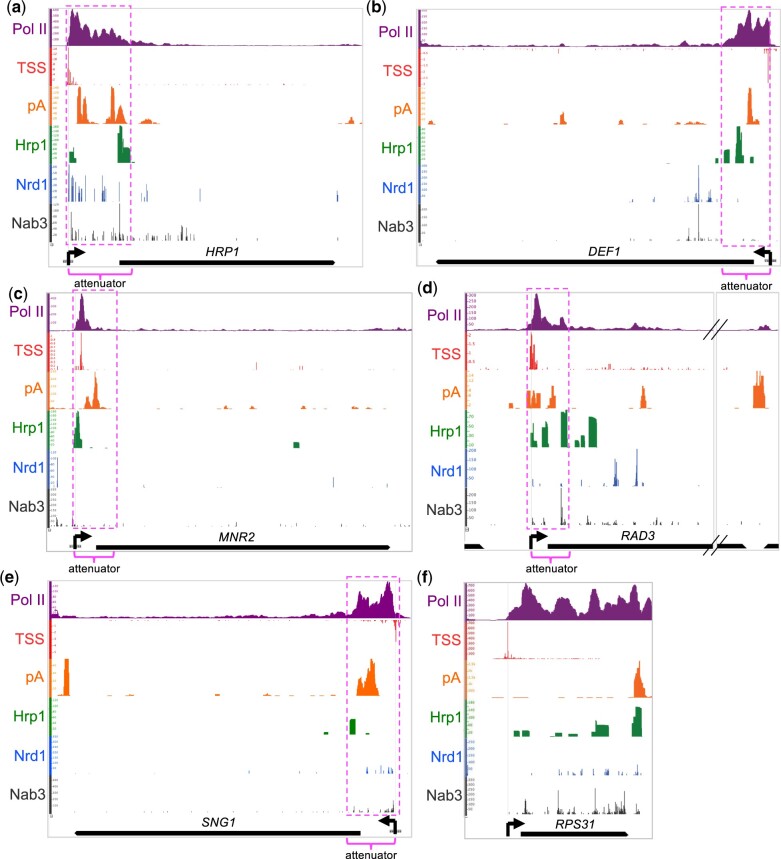
IGB analysis of Hrp1 and pA occupancy at promoter-proximal regions. a–e) For genes with known and putative attenuators, the protein occupancy [Pol II (Rpb1), Hrp1, Nrd1, Nab3], TSS, and polyadenylation (pA) sites were aligned to the *S. cerevisiae* genome and visualized with the IGB. Horizontal axes indicate genomic coordinates, and vertical axes display relative factor/site levels. Open reading frames and transcription directionality (left or right; black arrows) are indicated. Putative attenuators (fuchsia dashed lines) are based on 5′-end Pol II peaks and other contextual information. f) Comparative analysis was performed for the highly transcribed *RPS31* gene, with no evidence of attenuation.

Our analysis revealed intriguing localization patterns at established attenuators (*NRD1*, *HRP1*, *DEF1*) (Supplementary Fig. 6 and Fig. 6, a and b) as well as the identification of new putative Hrp1-dependent attenuators (*MNR2, RAD3, SNG1*) (Fig. 6, c–e). All of these genes exhibited common attenuation signatures: (1) upstream (5′-end) peak of Pol II with reduced signal throughout the open reading frame; (2) promoter-proximal pA sites; and (3) an upstream Hrp1 peak. In contrast, *RPS31* serves as an example of a highly transcribed gene without these attenuation signatures (Fig. 6f). *RPS31* exhibits high Pol II occupancy distributed relatively evenly across the entire open reading frame, and the highest levels of pA and Hrp1 signal overlap at the downstream (3′ end) of the gene.

**Fig. 7.**
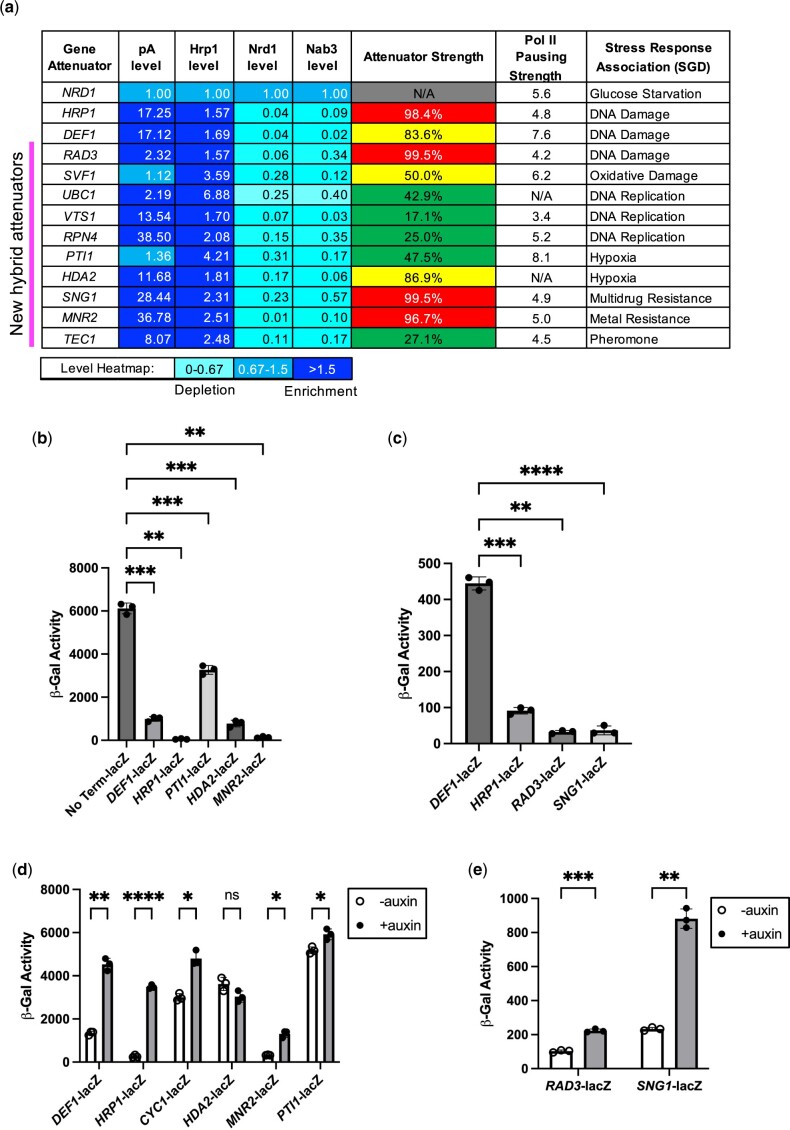
Heatmap analysis of Hrp1 and pA promoter-proximal enrichment helps identify new attenuators, some of which are Hrp1-dependent. a) The pA signals and protein factor occupancy from published studies were used to generate a heatmap. Levels were normalized to local Pol II attenuator peaks to account for differences in transcription rate and then to the canonical NNS-dependent *NRD1* attenuator. Blue shading indicates 1.5-fold enrichment or depletion relative to the *NRD1* attenuator. Attenuators were cloned into the lacZ reporter gene, and terminator strength was determined by the decrease in β-gal signal compared with a control reporter plasmid lacking a terminator (red >90%; yellow >50%; green <50%). Pol II pausing strength and stress response association were taken from Cherry *et al.* (2012) and Collin *et al.* (2019), respectively. b, c) Representative lacZ data for candidate attenuators after a 1- or 2-h kinetic assay. A lacZ reporter lacking a terminator (No Term-lacZ) serves as a negative control. d, e) The Hrp1 degron protein was depleted as described in Fig. 5f, followed by detection of β-galactosidase activity. Asterisks indicate statistical significance by Welch’s 2 sample *t*-test.

Given our interest in the Hrp1/Sen1 hybrid termination pathway, we developed a system to focus on genes with an early Pol II peak aligned with enrichment of pA sites and Hrp1 but little if any Nrd1/Nab3. To account for differences in gene transcription rate, we normalized pA signals and protein factor occupancy of Hrp1, Nrd1, and Nab3 to local Pol II levels. We then normalized these ratios to the 5′ end of *NRD1*, the canonical example of a Nrd1/Nab3-dependent attenuator. Based on these criteria, we identified 10 putative attenuators with >1.5-fold enrichment of pA/Hrp1 and >1.5-fold depletion (0–0.67) of Nrd1/Nab3 relative to *NRD1* (Fig. 7a). Each of the 10 putative attenuators exhibited terminator activity in a lacZ reporter gene assay, repressing Pol II readthrough (i.e. β-galactosidase activity) from 17.1% to 99.5% (Fig. 7, a–c). The *MNR2*, *RAD3*, and *SNG1* gene attenuators rank among the strongest identified to date, repressing Pol II readthrough at levels similar to or in excess of the *HRP1* attenuator. Interestingly, the 10 putative attenuator genes have connections to yeast cell stress responses (e.g. DNA damage, hypoxia, drug resistance, metal resistance, and pheromone) (Fig. 7a).

To examine whether the putative new attenuator candidates were Hrp1-dependent, we introduced some of the attenuator-lacZ reporter genes into the Hrp1 degron strain and compared the β-gal activity±auxin. The *DEF1*, *CYC1*, and *HRP1* reporters exhibited more β-gal activity after auxin treatment, consistent with Hrp1 degradation leading to Pol II readthrough of the attenuator (Fig. 7d). Likewise, the *MNR2*, *RAD3*, and *SNG1* attenuators were also Hrp1-dependent based on the observed β-gal increase±auxin (>1.5-fold) (Fig. 7, d and e). In contrast, there was minimal if any effect of Hrp1 depletion on reporter gene activity for the *HDA2* or *PTI1* attenuators (Fig. 7d**)**, despite their attenuation signatures (Supplementary Fig. 6, b and c). These results indicate that genome heatmap analysis can be a useful way to identify new attenuator candidates, but more criteria are needed to precisely determine the gene subset requiring Hrp1 activity.

### The readthrough defect of the hrp1-F162W RRM1 mutant extends to multiple attenuators and for some exhibits a strong dominant-negative phenotype

**Fig. 8.**
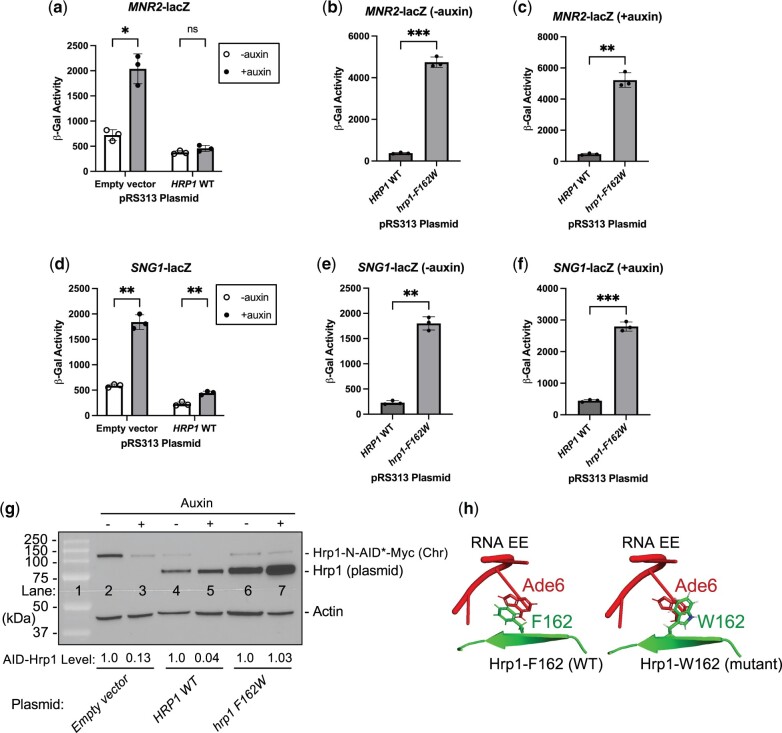
The *hrp1-F162W* RRM1 mutant exhibits strong dominant-negative readthrough defects at a subset of Hrp1-dependent attenuators. The Hrp1 degron strain containing (a–c) *MNR2*- or (d–f) *SNG1*-lacZ reporter genes was transformed with plasmids containing empty vector, *HRP1* WT, or mutant *hrp1-F162W*. Auxin treatment and β-gal assays were performed as in Fig. 5f. Asterisks indicate statistical significance by Welch’s 2 sample *t*-test or ANOVA. g) Western blot analysis of Hrp1 depletion ± auxin and Hrp1 plasmid-based expression. Following auxin treatment, the same *MNR2*-lacZ cultures used in (a) were used to harvest cells for protein extracts. AID-Hrp1 and untagged Hrp1 were detected with anti-Hrp1 antibody, and actin serves as a loading control. h) Protein–RNA interface of Hrp1-EE complex (structure 2KM8; PyMOL), highlighting Hrp1-F162 (WT) and RNA–A6 interaction. Hrp1 side chains (green) and RNA bases (red) represented in stick format, and the W162 mutant modeled using mutagenesis tool (PyMol).

Based on our earlier identification of the Hrp1 RRM residue F162 being important for attenuator recognition, we expanded our analysis to include the *MNR2* and *SNG1* attenuators. We again monitored lacZ reporter activity in Hrp1 degron strains containing empty vector or plasmid-based copies of *HRP1* wild-type and *hrp1*-*F162W*. As expected, the Hrp1 degron strain with empty vector exhibited readthrough defects for *MNR2* and *SNG1* ± auxin (∼3-fold), and plasmid addition of *HRP1* wild-type improved attenuator recognition (Fig. 8, a and d). Surprisingly, the *hrp1-F162W* mutant exhibited strong dominant-negative effects for both *MNR2* and *SNG1* attenuators even without Hrp1 depletion, increasing lacZ activity ∼13- and ∼8-fold compared with *HRP1* wild-type (Fig. 8, b and e). The addition of auxin further exacerbated the F162W readthrough defect for both *MNR2* and *SNG1* attenuators (Fig. 8, c and f).

Analysis of protein levels via western blot revealed that auxin treatment effectively depleted Hrp1-N-AID*-Myc protein ∼8-fold when expressed from the chromosomal locus (Fig. 8g; compare lanes 2 and 3). The addition of plasmid-based *HRP1* reduced Hrp1-N-AID*-Myc protein expression even in the absence of auxin (Fig. 8g; compare lanes 2 and 4), perhaps due to *HRP1* autoregulation (see *Discussion*), but auxin treatment was still effective in depletion (Fig. 8g; compare lanes 4 and 5). The plasmid-based *hrp1-F162W* mutant was stably expressed at levels even higher than Hrp1 WT (Fig. 8g; compare lanes 4 and 6), supporting a mutant defect that was not due to general protein instability. The Hrp1-N-AID*-Myc protein was not depleted during auxin treatment in the *hrp1-F162W* mutant (Fig. 8g; compare lanes 6 and 7), consistent with an *hrp1-F162W* dominant-negative effect and a compensatory mechanism of Hrp1 expression. Molecular modeling suggests that the *hrp1-F162W* mutant alters the base-stacking properties of the Hrp1 RRM with its target Ade6 EE RNA nucleotide (Fig. 8h). Overall, these results expand the importance of the Hrp1 RRM1 residue F162 in attenuator recognition and suggest a mechanism for RNA binding at a new class of attenuators.

## Discussion

Transcription attenuation of RNA polymerase has long been known as a significant regulatory mechanism in bacteria, where it provides a prompt and adaptable response to changing environmental conditions (Turnbough 2019). Transcription attenuation of eukaryotic Pol II is a more recently appreciated phenomenon, but numerous questions remain regarding its prevalence and underlying process. In this study, we further support the biological significance of attenuation in the yeast model eukaryote, confirming its importance in controlling the expression of the DNA damage response gene *DEF1*. We further establish that the 3′-end processing protein Hrp1 serves as a bona fide attenuator factor, with a crucial function for Hrp1 amino acid residues that bind CFIA protein Rna14 and RNA. We also identify new targets of Hrp1-dependent attenuation, expanding the role of hybrid termination as a mechanism for controlling eukaryotic mRNA expression.

### Expanding the biological significance of Pol II attenuation in eukaryotes

In this study, we demonstrate that the yeast *DEF1* attenuator contributes to biologically meaningful regulation at its natural genomic locus. This finding places the *DEF1* gene in exclusive company since relatively few eukaryotic attenuators have been tested for a role in biological fitness despite their ever-increasing identification. One such example attenuator is located in the *PCF11* gene, which encodes a 3′-end processing/termination factor that autoregulates its own expression (Creamer *et al.* 2011; Grzechnik *et al.* 2015; Kamieniarz-Gdula *et al.* 2019; Wang *et al.* 2019). In yeast, recognition of the *PCF11* attenuator is mediated by the NNS termination pathway (Creamer *et al.* 2011; Grzechnik *et al.* 2015). In higher eukaryotes, *PCF11* attenuation relies on the usage of a pA site located in an upstream intron, presumably via CPF-CF recognition (Kamieniarz-Gdula *et al.* 2019; Wang *et al.* 2019). Similar to our findings for the *DEF1* attenuator, deletion of the *PCF11* attenuator in zebrafish resulted in ∼2-fold upregulation of its product mRNA and protein (Kamieniarz-Gdula *et al.* 2019). Interestingly, this Pcf11 overexpression severely impacted vertebrate development, leading to embryonic death. Deletion of the human *PCF11* autoregulatory pA site in 4T1 metastatic cells slowed cell migration and invasion rates by 70%, suggesting a role for *PCF11* in cancer development (Wang *et al.* 2019).

In addition to *DEF1*, another example of biologically meaningful attenuation was discovered for the yeast *GLT1* gene, which encodes glutamine synthetase. Depletion of the NNS protein Nab3 led to readthrough of the *GLT1* attenuator, upregulating glutamine synthetase expression and increasing resistance to a glutamine synthetase inhibitor (Merran and Corden 2017). Among the newly identified Hrp1-dependent attenuators in this study, yeast overexpression of *MNR2* and *SNG1* is known to be toxic (Sopko *et al.* 2006; Yoshikawa *et al.* 2011), consistent with attenuation being important for negative regulation of these genes. It is also notable that *RAD3* contributes to nucleotide excision repair (Prakash and Prakash 2000), which given the function of *DEF1* suggests that attenuation may coordinately regulate some DNA repair genes. A genome-wide search for additional Hrp1 targets, particularly in cell stress genes, will help uncover features shared for functional regulation via hybrid termination.

Compared with the examples above, a seemingly unique quality of *DEF1* attenuation is that it operates in parallel with posttranslational regulation that controls Def1 cytoplasmic vs nuclear localization (Wilson *et al.* 2013). Why might attenuator-based regulation be beneficial for a yeast cell, aside from fine-tuning *DEF1* expression? We speculate that following UV stress, the yeast cytoplasmic Def1 pool is rapidly depleted due to nuclear import and the Def1 function in DNA repair. Upregulation of *DEF1* transcription could be necessary to quickly restore the cytoplasmic Def1 pool, either for a future DNA damage response and/or an additional Def1 function outside the nucleus (Akinniyi and Reese 2021). Once the genotoxic stress has passed, attenuation could facilitate *DEF1* transcriptional shut-off similar to what has been proposed for MtnA regulation by the Integrator complex during copper stress (Tatomer *et al.* 2019).

### The 3′-end processing factor Hrp1 is a gene-specific and RRM-dependent transcriptional regulator that also functions at 5′-end gene sequences

Hrp1 was initially identified as a 3′-end processing factor, which serves to bind the pA site EE and provide a scaffold for the cleavage/polyadenylation complex (Kessler *et al.* 1997). More recently Hrp1 has been implicated in Pol II attenuation at the 5′ end of genes (Steinmetz *et al.* 2006; Kuehner and Brow 2008; Tuck and Tollervey 2013; Chen *et al.* 2017; Whalen *et al.* 2018). In this work, we clarify the molecular role of Hrp1 during attenuator recognition by exploring the importance of its CFIA interaction and RRM. The *hrp1-L205S* mutant was previously shown to be temperature sensitive, impair 3′-end processing, and disrupt Rna14 interaction (Kessler *et al.* 1997; Barnwal *et al.* 2012). In this study, the *hrp1-L205S* mutant caused readthrough of the *CYC1* terminator as well as *DEF1* and *HRP1* attenuators, supporting a potential role for Hrp1–Rna14 interaction in both 3′-end processing/termination and attenuator recognition. The *hrp1-D193N* mutant proposed to disrupt binding with Rna15 (Leeper *et al.* 2010) did not affect termination for our reporter genes. Given that we previously observed strong readthrough of the *DEF1* attenuator in an *rna15-1* mutant (Whalen *et al.* 2018), additional exploration is needed to gauge the importance of Hrp1-Rna15 and the CFIA complex in hybrid termination at promoter-proximal gene locations.

To test the importance of the Hrp1 RRM in attenuation we first assayed the temperature-sensitive *hrp1-K160E* (*hrp1-5*) RRM1 mutant (Kessler *et al.* 1997), which caused modest readthrough (1.5-fold) of the control *CYC1* terminator but not *DEF1* or *HRP1* attenuators. The *hrp1-K160E* mutant was proposed to reduce RNA recognition since K160 potentially forms a hydrogen bond with the Ura7 base of EE RNA (Perez-Canadillas 2006). However, others have argued that Hrp1-K160 provides more indirect and passive RNA interaction (Barnwal *et al.* 2012). We identified new *hrp1* mutant alleles in RRM1 that conferred readthrough defects at both the *CYC1* control terminator and multiple attenuators, with the most substantial impact for *hrp1-F162W*. A phenylalanine at the position equivalent to F162 of yeast Hrp1 is conserved in protein RNA-binding domains from numerous other species, including multicellular organisms such as insects, plants, and mammals as well as human HNRNPDL (Kamei *et al.* 1999; Perez-Canadillas 2006). In the yeast Hrp1:RNA structure, the aromatic R-group of F162 forms stacking interactions with an Ade6 base of the EE (Perez-Canadillas 2006; Barnwal *et al.* 2012). Our molecular modeling suggests that the W162 mutant may be altered with respect to the RNA base, disrupting the interaction and leading to readthrough. The extent of Hrp1-dependent attenuation was gene-specific, with attenuators either demonstrating no sensitivity (*HDA2*, *PTI1*), moderate sensitivity (*DEF1*), or strong sensitivity (*SNG1*, *MNR2*, *HRP1*) to Hrp1 depletion.

Another interesting feature of the *hrp1-F162W* mutant was its dominant-negative behavior (Veitia 2009). Attenuator defects were observed in the *hrp1-F162W* strain even in the presence of functional Hrp1. The readthrough defect appears to be at least partly due to higher plasmid-based expression of *hrp1-F162W* compared with chromosomal *HRP1-N-AID*. The higher expression of the mutant protein may be caused by plasmid amplification and/or readthrough of the *HRP1* autoregulatory attenuator due to an *hrp1-F162W* defect. Consistent with this model, the *hrp1-F162W/HRP1-N-AID* strain grows more slowly than *HRP1-N-AID* (data not shown), and Hrp1-N-AID fails to be depleted after auxin treatment in the *hrp1-F162W* strain. Variation in the degree of dominant-negative readthrough likely reflects attenuator sequence context, which may correspond to changes in the number of EE RNA motifs and Hrp1-binding sites. In the case of dominant-negative mutations in transcription factors, mutations have been identified that disrupt nucleic acid binding but not dimerization (Veitia 2009). There is some evidence to suggest that Hrp1 may bind cooperatively to longer EE elements, including a 2:1 Hrp1:RNA stoichiometry in chemical shift perturbation experiments as well as higher molecular weight complexes at high protein/RNA ratios (Perez-Canadillas 2006). In the future, it will be helpful to characterize the *cis*-acting sequences required for Hrp1-dependent attenuator recognition and if attenuators highly sensitive to the *hrp1-F162W* mutant contain unique tandem EE arrangements.

## Supplementary Material

jkac292_Supplementary_Data

## Data Availability

Strains, plasmids, and primer sequences are available upon request. Supplementary Tables 1–3 contain detailed descriptions of all yeast strains, primers, and plasmids used in this study. Data used to generate IGB plots in Fig. 6 and Supplementary Fig. 6 are available at GEO with the accession numbers: GSE39128 (TSS), GSE30706 (pA), GSE56435 (Pol II), GSE46742 (Hrp1), and GSE31764 (Nab3, Nrd1). Supplemental material is available at G3 online.
