# Supplementary material for: RNA polymerase II transcription attenuation at the yeast DNA repair gene DEF1 is biologically significant and dependent on the Hrp1 RNA-recognition motif
Source: G3 (Bethesda). 2022 Oct 31;13(1):jkac292. doi: 10.1093/g3journal/jkac292 (PMC9836349; doi:10.1093/g3journal/jkac292)
Supplement: jkac292_Supplementary_Data [file jkac292_supplementary_data.zip › Suppl/Table_S3_G3-2022-403884.docx]

**Table S3. Plasmids used in this study**

| **Plasmid Name** | **Description** | **Selectable Marker** | **Source** |
| --- | --- | --- | --- |
| p416-TEF1p-Cas9pCYC1t-crRNA-BaeI | Contains *S. pyogenes* Cas9 and a cloning site (BaeI) for sgRNA flanked by SNR52 promoter and gRNA scaffold | *AMP^R^, URA3* | Talkish *et al.* 2019 |
| p416-Cas9, DEF1 531 stop | CRISPR plasmid containing guide RNA to create pr-Def1 | *AMP^R^, URA3* | This study |
| p416-Cas9, DEF1 hrp1 sub (TATATA🡪GCGACG) | CRISPR plasmid containing guide RNA to disrupt *DEF1* attenuator | *AMP^R^, URA3* | This study |
| p416-Cas9, HRP1-N-AID | CRISPR plasmid containing guide RNA to disrupt *DEF1* attenuator | *AMP^R^, URA3* | This study |
| pKan-*PCUP1-9myc-AID*(N)* | Contains *CUP1* promoter, auxin-inducible degron + Myc for N-terminal tagging | *AMP^R^, KANMX* | Morawska *et al.* 2013 |
| pKan-*AID*-9myc* | Contains auxin-inducible degron + Myc for C-terminal tagging | *AMP^R^, KANMX* | Morawska *et al.* 2013 |
| pGPD1-*osTIR1*, pTIR4 | Contains *O. sativa* E3 ligase *TIR1* expressed from *GPD1* promoter, which is necessary for AID degradation; integrates at *LEU2* locus | *AMP^R^, LEU2* | Nishimura *et al.* 2009 |
| pRS313 | A low copy centromere shuttle vector | *HIS3* | Sikorski 1989 |
| pRS313-*HRP1* | Contains *HRP1* promoter and open reading frame (-500 to +1848 relative to the *HRP1* +1 ATG) | *AMP^R^, HIS3* | Whalen *et al.* 2018 |
| pRS313-*hrp1 K160E, W168A, F162W, W168F, D193N, F204W, or L205S* | Contains *HRP1* promoter and open reading frame (-500 to +1848 relative to the *HRP1* +1 ATG) plus the amino acid substitution indicated | *AMP^R^, HIS3* | This study |
| pGAC24-noTerm-lacZ | Contains *ACT1* exon-intron-exon fused to lacZ, XhoI intron cloning site, no terminator present |  | Whalen *et al.* 2018 |
