# Supplementary material for: RNA polymerase II transcription attenuation at the yeast DNA repair gene DEF1 is biologically significant and dependent on the Hrp1 RNA-recognition motif
Source: G3 (Bethesda). 2022 Oct 31;13(1):jkac292. doi: 10.1093/g3journal/jkac292 (PMC9836349; doi:10.1093/g3journal/jkac292)
Supplement: jkac292_Supplementary_Data [file jkac292_supplementary_data.zip › Suppl/Supplemental_Figure_Legends_G3-2022-403884.docx]

**Supplemental Figure Legends**

**Figure S1. The *DEF1* attenuator mutant (*def1_atten_*) overexpresses mRNA and protein compared to *DEF1* wild-type.**

(A) RT-PCR analysis of Def1 mRNA levels in *DEF1* wild-type and *def1_atten_* mutant. 18S serves as a loading control for total RNA. Reverse Transcriptase (-/+RT) ensures that signal is dependent on RNA and not genomic DNA template.

(B) Western blot analysis of Def1 protein levels in *DEF1* wild-type and *def1_atten_* mutant, performed in biological triplicate. Actin serves as a loading control for total loaded protein.

(C) The average Def1 protein levels (normalized to actin) were quantified from the 3 biological replicates in (B), and error bars represent the standard deviation. Asterisks indicate statistical significance by Welch’s two sample t-test.

**Figure S2. The *hrp1-W168F* mutant mildly suppresses attenuator readthrough in the *DEF1*, *CYC1* and *HRP1-LacZ* reporter genes.**

Attenuator functionality assays using a lacZ reporter gene with *HRP1* wild-type and *hrp1-W168F* mutant strains. A yeast shuffle strain [*hrp1::KANMX*, pRS316-*HRP1* (*URA3*)] was transformed with *HIS3*-marked plasmids containing *HRP1* WT or *hrp1* mutants prior to 5-FOA shuffling, followed by transformation with lacZ reporter genes containing *DEF1* or *HRP1* attenuators. The *CYC1* terminator serves as a control for hybrid termination. Overnight cultures were grown to saturation at 30°C and recovered to exponential phase followed by a 2-hour shift to elevated temperature (37°C). Cells were lysed and β-galactosidase activity was measured to detect attenuator read-through. Error bars represent standard deviation of 3 biological replicates. Asterisks indicate statistical significance by Welch’s two sample t-test.

**Figure S3. The *HRP1-C-AID*-Myc* allele is defective even in the absence of auxin.**

(A) Yeast strains containing WT (*HRP1)*, N-terminal (*HRP1-N-AID*-Myc*) or C-terminal degron tags (*HRP1-C-AID*-Myc*) were grown on YPAD -/+ auxin inducer (1 mM) for 3 days at the indicated temperatures.

(B) Yeast strains bearing *HRP1* WT, *TIR1*, *HRP1-C-AID-Myc*, or a combination of each were transformed with the *DEF1*-lacZ reporter gene. Cultures were grown overnight in selective media, cells were diluted for recovery and lysed, and β-galactosidase activity was measured to detect attenuator read-through. Error bars represent standard deviation of 3 biological replicates. Asterisks indicate statistical significance by Welch’s ANOVA.

**Figure S4. Auxin addition to strains lacking an AID-tagged Hrp1 does not cause attenuator readthrough of lacZ reporter genes.**

The BY4742 wild-type strain was transformed with indicated lacZ reporter genes and grown in selective media -/+ auxin (4 mM) for 4 hours. Cells were lysed and β-galactosidase activity was measured to detect attenuator read-through. Error bars represent standard deviation of 3 biological replicates. Statistical significance was tested by a Welch’s two sample t-test.

**Figure S5. The *CYC1-lacZ* reporter exhibits Pol II readthrough defects in *hrp1* RRM mutants W168A, F162W, and F204W.**

The Hrp1 degron strain (*HRP1-N-AID*-Myc*) was transformed with the *CYC1-lacZ* reporter gene and grown in selective media -/+ auxin for 4 hours. Cells were lysed and β-galactosidase activity was measured to detect Pol II terminator read-through. Error bars represent standard deviation of 3 biological replicates. The horizontal dashed line serves as a reference point for lacZ activity in the *HRP1* wild-type +auxin strain. Asterisks indicate statistical significance by Welch’s two sample t-test.

**Figure S6. Integrated Genome Browser analysis of Hrp1 and pA occupancy at *NRD1*, *PTI1*, and *HDA2* promoter-proximal regions.**

The protein occupancy (Pol II (Rpb1), Hrp1, Nrd1, Nab3), transcription start sites (TSS), and polyadenylation (pA) sites were aligned to the *S. cerevisiae* genome and visualized with the Integrated Genome Browser. Horizontal axes indicate genomic coordinates, and vertical axes display relative factor/site levels. The open reading frame and transcription directionality (left or right; black arrows) is indicated. The putative attenuator (fuchsia dashed lines) is based on 5’-end Pol II peaks and other contextual information.
