# Supplementary material for: RNA polymerase II transcription attenuation at the yeast DNA repair gene DEF1 is biologically significant and dependent on the Hrp1 RNA-recognition motif
Source: G3 (Bethesda). 2022 Oct 31;13(1):jkac292. doi: 10.1093/g3journal/jkac292 (PMC9836349; doi:10.1093/g3journal/jkac292)
Supplement: jkac292_Supplementary_Data [file jkac292_supplementary_data.zip › Suppl/Table_S1_G3-2022-403884.docx]

**Table S1. Yeast strains used in this study**

| **Strain** | **Genotype** | **Source** |
| --- | --- | --- |
| BY4742 | *MATα his3Δ1 leu2Δ0 lys2Δ0 ura3Δ0* | Brachmann 1998 |
| BY4742 def1_1-530_ | *MATα his3Δ1 leu2Δ0 lys2Δ0 ura3Δ0 def1_1-530_* | This study |
| BY4742 def1_atten_ | *MATα his3Δ1 leu2Δ0 lys2Δ0 ura3Δ0 def1_atten_* | This study |
| BY4742 def1_1-530+atten_ | *MATα his3Δ1 leu2Δ0 lys2Δ0 ura3Δ0 def1_1-530+atten_* | This study |
| BY4742 *HRP1* shuffle strain | *MATα his3Δ1 leu2Δ0 lys2Δ0 ura3Δ0 hrp1::KANMX + pRS316-HRP1 (URA3)* | This study |
| BY4742 *HRP1* shuffle strain + viable *hrp1* mutants | *MATα his3Δ1 leu2Δ0 lys2Δ0 ura3Δ0 hrp1::KANMX + pRS313-hrp1 K160E, W168F, D193N, or L205S (HIS3)* | This study |
| BY4742 auxin-inducible degron starter strain | *MATα his3Δ1 leu2Δ0 lys2Δ0 ura3Δ0 osTIR1::LEU2* | This study |
| BY4742 *HRP1* *HRP1-N-AID* | *MATα his3Δ1 leu2Δ0 lys2Δ0 ura3Δ0 osTIR1::LEU2 HRP1-9Myc-AID*(N)* | This study |
| BY4742 *HRP1-N-AID* + lacZ attenuator reporter | *MATα his3Δ1 leu2Δ0 lys2Δ0 ura3Δ0 osTIR1::LEU2 HRP1-9Myc-AID*(N)*  + pGAC24-*CYC1*-lacZ, *DEF1*-lacZ, *HRP1*-lacZ, or *SNR13*-lacZ (*LEU2*) | This study |
| BY4742 *HRP1-C-AID* | *MATα his3Δ1 leu2Δ0 lys2Δ0 ura3Δ0 osTIR1::LEU2 HRP1-AID*-9Myc* | This study |
| BY4742 *HRP1-N-AID* + lethal hrp1 mutants | *MATα his3Δ1 leu2Δ0 lys2Δ0 ura3Δ0 osTIR1::LEU2 HRP1-9Myc-AID*(N)*  + pRS313-empty, pRS313-*HRP1*, pRS313-*hrp1-W168A*, *F162*W, or *F204W* (*HIS3*) | This study |
| BY4742 *HRP1-N-AID* + lethal hrp1 mutants + lacZ attenuator reporter | *MATα his3Δ1 leu2Δ0 lys2Δ0 ura3Δ0 osTIR1::URA3 HRP1-9Myc-AID*(N)*  *+* pRS313-empty, pRS313-HRP1, pRS313-*hrp1-F162W* (*HIS3*)  + pGAC24-*CYC1*-lacZ, *DEF1*-lacZ, *HRP1*-lacZ, *MNR2*-lacZ or *SNG1*-lacZ (*LEU2*) | This study |
