# Supplementary material for: RNA polymerase II transcription attenuation at the yeast DNA repair gene DEF1 is biologically significant and dependent on the Hrp1 RNA-recognition motif
Source: G3 (Bethesda). 2022 Oct 31;13(1):jkac292. doi: 10.1093/g3journal/jkac292 (PMC9836349; doi:10.1093/g3journal/jkac292)
Supplement: jkac292_Supplementary_Data [file jkac292_supplementary_data.zip › Suppl/Table_S2_G3-2022-403884.docx]

**Table S2. Primers used in this study**

| **Primer Name** | **Primer sequence (5’-3’)** |
| --- | --- |
| **CRISPR cloning and repair DNA PCR** | |
| DEF1 sgRNA1 Gibson_F | gcagtgaaagataaatgatcCATATATATATAATGTCTACACAATTTgttttagagctagaaatagc |
| DEF1 sgRNA1 Gibson_R | gctatttctagctctaaaaAAATTGTGTAGACATTATATATATATGgatcatttatctttcactgc |
| DEF1 sgRNA2 Gibson_F | gcagtgaaagataaatgatcGATTCACAAGGATACGCTTAgttttagagctagaaatagc |
| DEF1 sgRNA2 Gibson_R | gctatttctagctctaaaacTAAGCGTATCCTTGTGAATCgatcatttatctttcactgc |
| DEF1 Hrp1 Sub_F1 | CATCATAAAGTACTTACATATTTATTTTTGTTTGGTCGTTTTCTCAATATAATCTACATC |
| DEF1 Hrp1 Sub_R1 | AGCGTATCTATTTTGGACTTTAGCGCAGGATTTAGTTTTTTACTACTATGACTATTATGA |
| DEF1 Hrp1 Sub mut | AATCTACATCATCATATACGCACGATGTCTACACAATTTAGAAAGTCTAATCATAATAGT |
| DEF1 531 stop_F | GCTGCCCAACAGTACTACATGTATCAAAACCAATTTCCTGGATATTCTTATCCAGGTATG |
| DEF1 531 stop_R | CTTGTTGGAAATTATATTGGTTAGCATTACCACTAGTTTGAGCGTTGTTTTGAGCAAGTT |
| DEF1 531 stop mut | TCCAGGTATGTTTGATTCACAAGGATACTAATACGGTCAACAATATCAGCAACTTGCTCA |
| HRP1 sgRNA2 Gibson_F | gcagtgaaagataaatgatcGAAGATTTCAACGACATCTAgttttagagctagaaatagc |
| HRP1 sgRNA2 Gibson_R | gctatttctagctctaaaacTAGATGTCGTTGAAATCTTCgatcatttatctttcactgc |
| HRP1-MYC-AID-(N)_F primer | TACAATCTTTTCATAAAAGACGCAAATATTTTATTTATAAAGAACTTTAGCAGTATACACAAGTCTGAGAAAATAGAGAATAAGTTAAATAAGCAATGtccggttctgctgctagtggtg |
| HRP1-MYC-AID-(N)_R | ACCACTGCCAGCCTTATTTTGTTCTTCTTCTTTTTTGACTTCTTCAGTAGTGGTAGGCTTATCATCGCCGTATATATCATTGAAATCTTCTTCGTCAGAGCTcttcacgaacgccgccgc |
| HRP1 tag up | CAAGAAAACTTTTCTCTAGTTTTCTACACTTTTCTTTTTTTATCGATGAATTCGAGCTCG |
| HRP1 tag down | GGATACAATAGACGTAATAATGGCTACCATCCATATAATAGGCGTACGCTGCAGGTCGAC |
| SNR52 Forward Seq | GATAATGTATGATTATGCTTTCAC |
| **RT-PCR** | |
| DEF1 (-62)_F | CTTACATATTTATTTTTGTTTGGTCGTTTT |
| DEF1 (+139)_R | CTTGAACTATATCAATTAAATCATCAC |
| 18S rRNA_F | ACTTTTCGAATCGCATGGCC |
| 18S rRNA_R | GAATCGAACCCTTATTCCCC |
| **Attenuator cloning and confirmation** | |
| ACT1 (+1)_F | ATGGATTCTGGTATGTTCTAGC |
| ACT1(+155)_R | TCGTGGTTATTACAGATCAGTC |
| HDA2 Atten  (-283) Gib_F | CACGGTCCCAATTGCTCGAGATTTCCTGAGAAAGTTCTC |
| HDA2 Atten  (-1) Gib_R | AAAGGTAAAGAGAAATCTCTCGAGGTCGATAGTATTGTATCTATTTTC |
| MNR2 Atten  (-225) Gib_F | CACGGTCCCAATTGCTCGAGTATGAACCTAGCCCAG |
| MNR2 Atten  (+116) Gib_R | AAAGGTAAAAGAGAAAATCTCTCGAGACAAACTTGTCCTTTCTTC |
| PTI1 Atten  (-201) Gib_F | CACGGTCCCAATTGCTCGAGATACTAGTACGATTGTTTGAG |
| PTI1 Atten  (+195) Gib_R | AAAGGTAAAAGAGAAATCTCTCGAGGAACAGTACACCGGTTAG |
| RAD3 Atten  (-139) Gib_F | CACGGTCCCAATTGCTCGAGAATAATAAATAAATATATATATATGTATATCTTAAAGTTG |
| RAD3 Atten  (+175) Gib_R | AAAGGTAAAAGAGAAATCTCTCGAGAGGCAATTGTGAGGGATAG |
| RPN4 Atten  (-264) Gib_F | CACGGTCCCAATTGCTCGAGAAGCCCGGGATGAGCAGC |
| RPN4 Atten  (+98) Gib_R | AAAGGTAAAAGAGAAATCTCTCGAGTGACTCGTAAACTGACTGTGACCTG |
| SNG1 Atten  (-262) Gib_F | CACGGTCCCAA TTGCTCGAGGAAAGCCGTGAGTTTGTTTG |
| SNG1 Atten  (+115) Gib_R | AAAGGTAAAAGAGAAATCTCTCGAGCAAATCGCTGAT TACTGTAC |
| SVF1 Atten  (-234) Gib_F | CACGGTCCCAATTCGAGAAAAGCGAAATGACAGCTG |
| SVF1 Atten  (+117) Gib_R | AAAGGTAAAAGAGAAATCTCTCGAGTCTGTAAGGCTTATTTTTTA |
| TEC1 Atten  (-112) Gib_F | CACGGTCCCAATTGCAGTACTTTTATAAATGTTTATGAAACAGGAG |
| TEC1 Atten  (+297) Gib_R | AAAGGTAAAAGAGAAATCTCTCGAGCTTCCTCGCTTGTCAGTG |
| UBC1 Atten  (-126) Gib_F | CACGGTCCCAATTGCTCGAGGATCGCGGGCGTATAGGTAAAG |
| UBC1 Atten  (+173) Gib_R | AAAGGTAAAAGAGAAATCTCTCGAGACTTCGATATCCACGACAAATTTG |
| VTS1 Atten  (-187) Gib_F | CACGGTCCCAATTGCTCGAGAGCAAATTGGCGATGAGATATCTAAC |
| VTS1 Atten  (+82) Gib_R | AAAGGTAAAAGAGAAATCTCTCGAGAAACGGCGCCTGGATGAG |
| ***HRP1* Cloning and Mutagenesis** | |
| HRP1 (-500)-Not1_F | TAAGCAGCGGCCGCTACGAGCAGCGCCGAGATTTC |
| HRP1 (1848)-Xho1_R | TAAGCACTCGAGTGTCTGCCTCTTACATTCTTGCGC |
| Hrp1 W168F QC_F | CAAGATGTTCATTGGTGGTCTGAATTTCGACACTACGGAAGA |
| Hrp1 W168F QC_R | TCTTCCGTAGTGTCGAAATTCAGACCACCAATGAACATCTTG |
| Hrp1 W168A QC_F | CAAGATGTTCATTGGTGGTCTGAATGCGGACACTACGGA |
| Hrp1 W168A QC_R | TTCCGTAGTGTCCGCATTCAGACCACCAATGAACATCTT |
| Hrp1 F162W QC_F | CTAAAGAAAGTTGCAAGATGTGGATTGGTGGTCTGAATTGGGAC |
| Hrp1 F162W QC_R | GTCCCAATTCAGACCACCAATCCACATCTTGCAACTTTCTTTAG |
| Hrp1 F204W QC_F | GGTAGATCTAGAGGGTTCGGTTGGTTATCTTTTGAAAAACCTTCT |
| Hrp1 F204W QC_R | CTAGAATTTTCAAAAGATAACCAACCGAACCCTCTAGATCTACC |
| HRP1 D193N QC_F | CGTCACTGATTTGAAAATCATGAAAAACCCTGCAACAGG |
| HRP1 D193N QC_R | CCTGTTGCAGGGTTTTTCATGATTTTCAAATCAGTGACG |
| HRP1 K160E QC_F | GATTTGTCTAAAGAAAGTTGCGAGATGTTCATTGGTGGTCTGA |
| HRP1 K160E QC_R | TCAGACCACCAATGAACATCTCGCAACTTTCTTTAGACAAATC |
| HRP1 L205S QC_F | GATCTAGAGGGTTCGGTTTCTCATCTTTTGAAAAACCTTCTA |
| HRP1 L205S QC_R | CTAGAAGGTTTTTCAAAAGATGAGAAACCGAACCCTCTAGATC |
