# Supplementary figures and images for: RNA polymerase II transcription attenuation at the yeast DNA repair gene DEF1 is biologically significant and dependent on the Hrp1 RNA-recognition motif
Source: G3 (Bethesda). 2022 Oct 31;13(1):jkac292. doi: 10.1093/g3journal/jkac292 (PMC9836349; doi:10.1093/g3journal/jkac292)

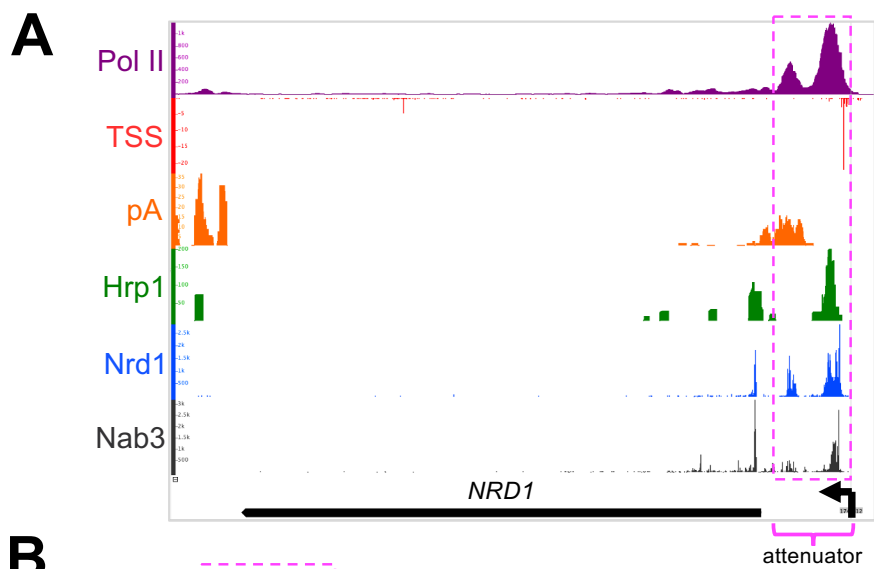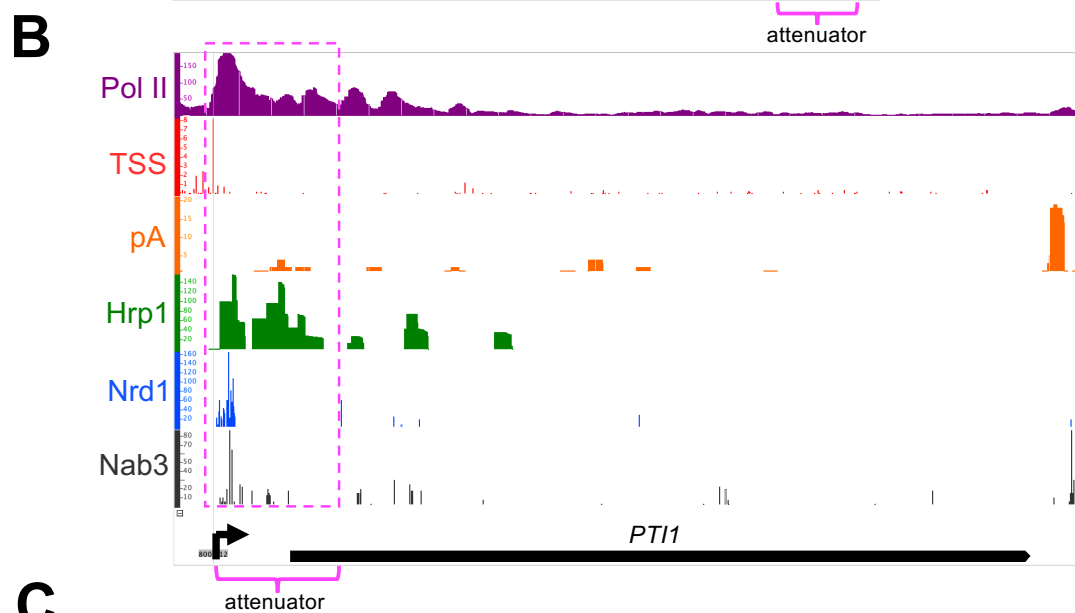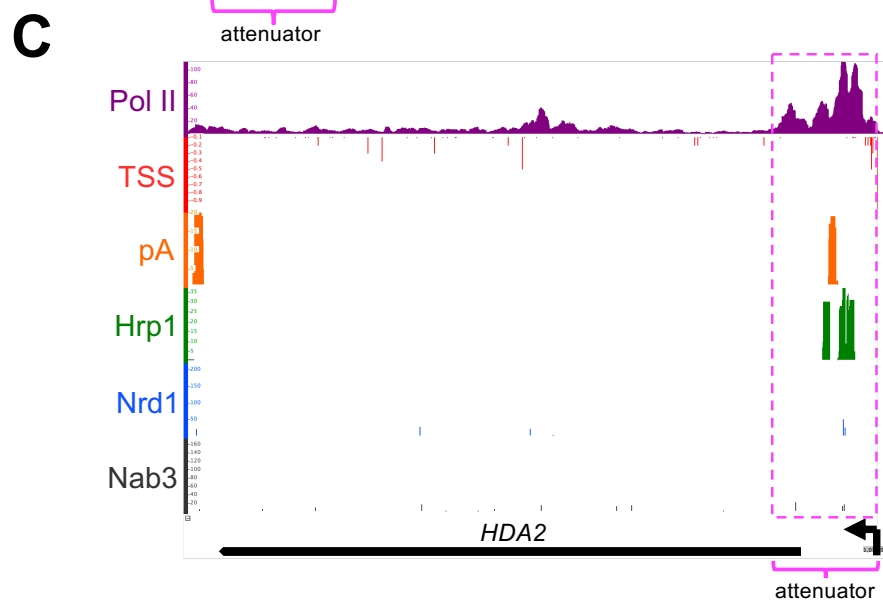

Supplement: jkac292_Supplementary_Data [file jkac292_supplementary_data.zip › Suppl/Figure_S6_G3-2022-403884.pdf]

**A**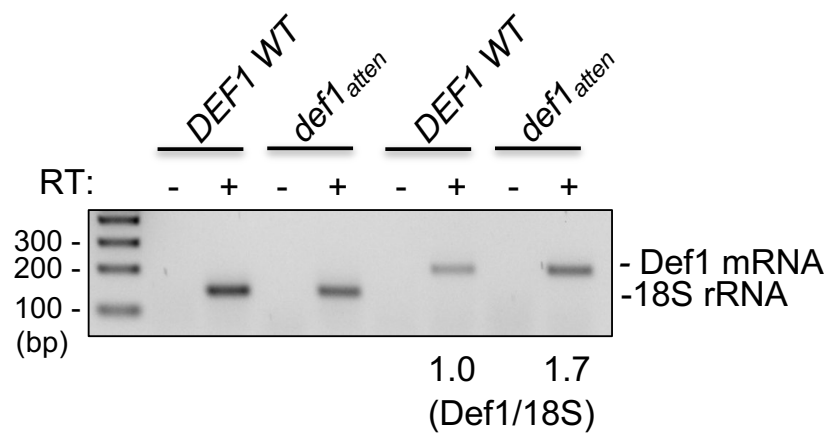**B**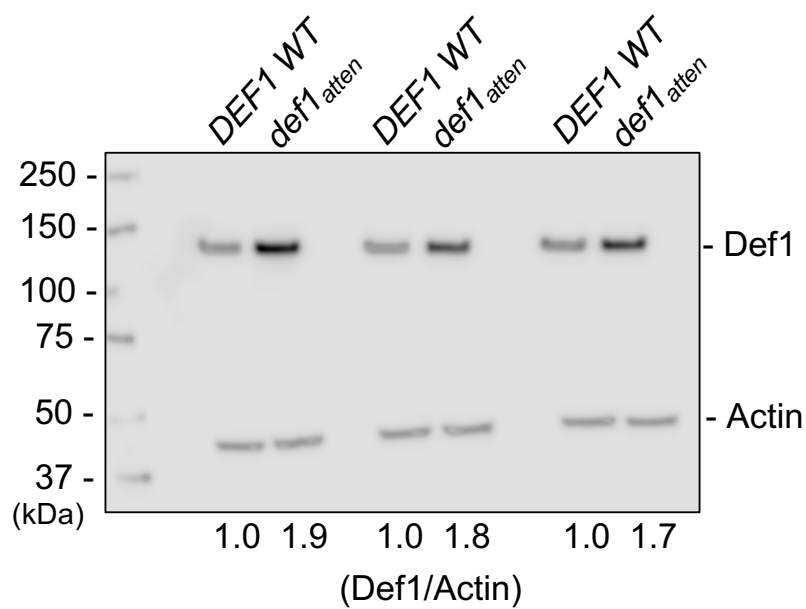**C**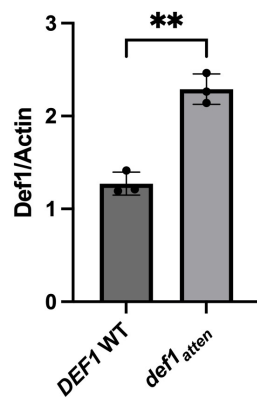

Supplement: jkac292_Supplementary_Data [file jkac292_supplementary_data.zip › Suppl/Figure_S1_G3-2022-403884.pdf]

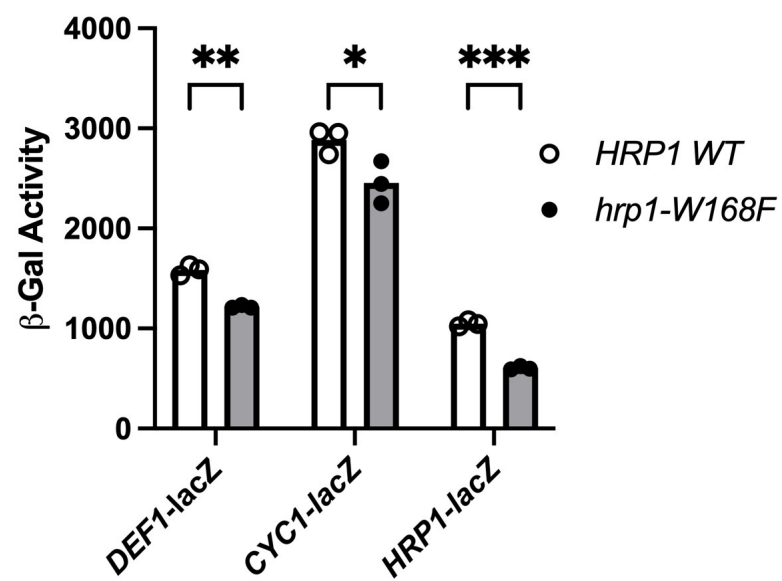

Supplement: jkac292_Supplementary_Data [file jkac292_supplementary_data.zip › Suppl/Figure_S2_G3-2022-403884.pdf]

# A

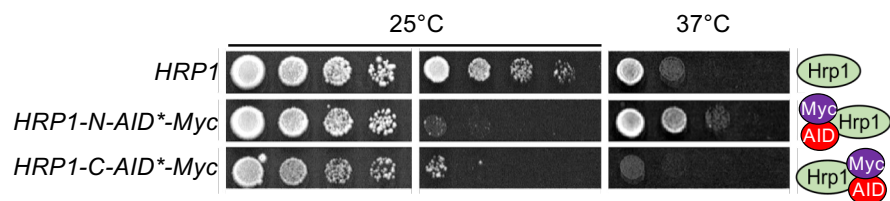

# B

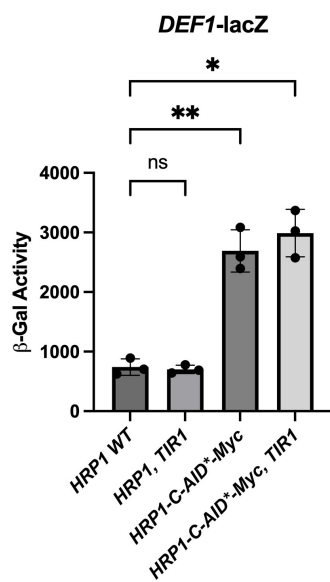

Supplement: jkac292_Supplementary_Data [file jkac292_supplementary_data.zip › Suppl/Figure_S3_G3-2022-403884.pdf]

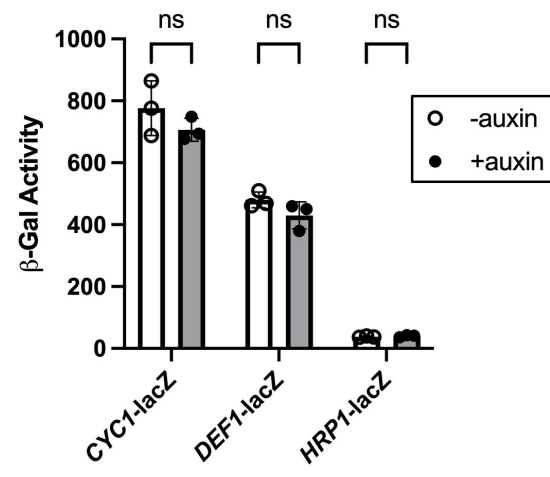

Supplement: jkac292_Supplementary_Data [file jkac292_supplementary_data.zip › Suppl/Figure_S4_G3-2022-403884.pdf]

**A**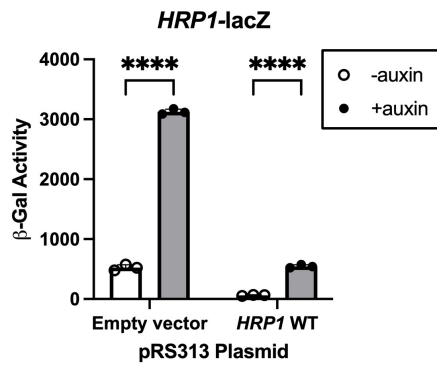**B**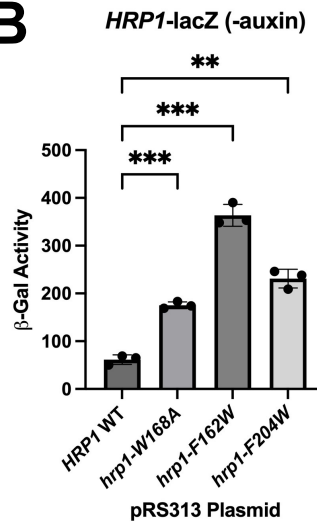**C**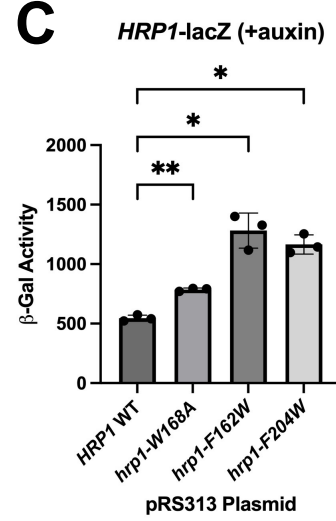**D**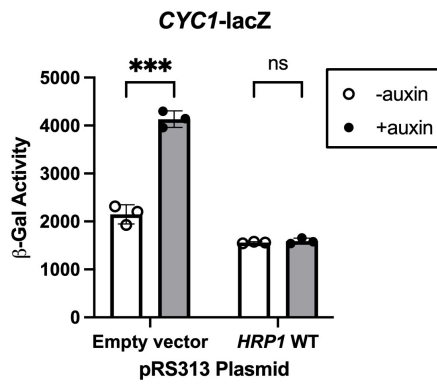**E**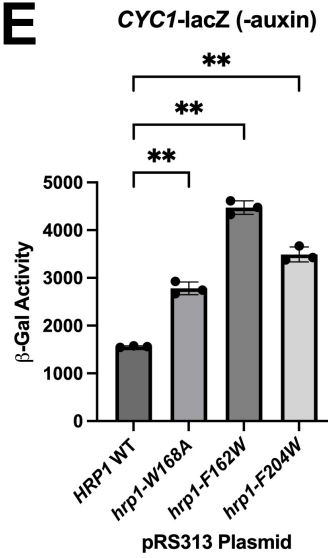**F**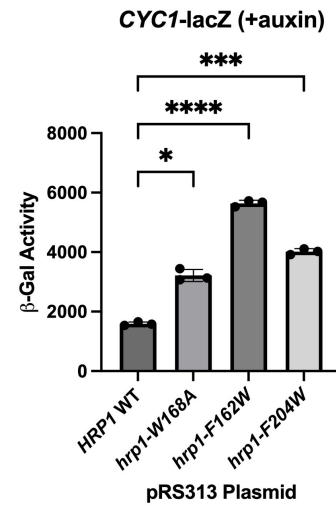

Supplement: jkac292_Supplementary_Data [file jkac292_supplementary_data.zip › Suppl/Figure_S5_G3-2022-403884.pdf]
